# Iso­propyl­ammonium halidometallates. I. [Co*X*_4_]^2−^·*X*^−^ (*X* = Cl, Br), ZnCl_4_^2−^, and [ZnCl_3_^−^]_*n*_ salts

**DOI:** 10.1107/S2056989025006103

**Published:** 2025-07-15

**Authors:** Josh Birke, Tu Nguyen, Marcus R. Bond

**Affiliations:** a Department of Chemistry and Physics Southeast Missouri State University,Cape Girardeau MO 63701 USA; National Taras Shevchenko University of Kyiv, Ukraine

**Keywords:** crystal structure, iso­propyl­ammonium, halidometallates, one-dimensional chains

## Abstract

An exploration of the iso­propyl­ammonium (IPA^+^) metal halide structural space is conducted, initially, for cobalt(II) and zinc(II) halides as a counterpart to the more extensively studied metal halide systems containing the isomeric tri­methyl­ammonium cation. For cobalt(II) halides, the only compound obtained from slow evaporation of acidic aqueous solutions of iso­propyl­ammonium halide and cobalt(II) halide is (IPA^+^)_3_Co*X*_4_·*X* (*X* = Cl, Br), namely, tris­(iso­propyl­ammonium) tetra­chlorido­cobaltate(II) chloride, (C_3_H_10_N)_3_[CoCl_4_]Cl, and tris­(iso­propyl­ammonium) tetra­bromido­cobaltate(II) bromide, (C_3_H_10_N)_3_[CoBr_4_]Br, regardless of the starting stoichiometric ratio of (IPA^+^)*X*:Co*X*_2_. These structures consist of isolated tetra­hedral Co*X*_4_^2−^ complexes and *X*^−^ ions separated by IPA^+^ cations. One IPA^+^ cation hydrogen bonds to two complexes and to two halide ions to produce clusters. These clusters form stacks with hydrogen bonding from the remaining IPA^+^ cations linking the stacks into a tight, three-dimensional network. A 2:1 molar ratio of (IPA^+^)Cl and ZnCl_2_ under similar growth conditions readily yields crystals of bis­(iso­propyl­ammonium) tetra­chlorido­zincate(II), (C_3_H_10_N)_2_[ZnCl_4_] or (IPA^+^)_2_ZnCl_4_, consisting only of IPA^+^ cations and isolated ZnCl_4_^2−^ tetra­hedra. This despite the expectation of common structural chemistry for the similarly sized Co^2+^ and Zn^2+^ ions with 51 known isostructural A_2_CoCl_4_ and A_2_ZnCl_4_ compounds out of 54 for the same monopositive A cation. Growth from a 1:1 ratio of (IPA^+^)Cl:ZnCl_2_ yields crystals of poly[iso­propyl­ammonium [[di­chlorido­zincate(II)]-μ-chlorido]], {(C_3_H_10_N)[ZnCl_3_]}_*n*_ or (IPA^+^)ZnCl_3_, in which parallel chains of corner-sharing ZnCl_4_ tetra­hedra are separated by IPA^+^ cations. This unusual zinc(II) halide chain structure has previously been observed only in [*Y*CH_2_(CH_3_)_3_N]ZnCl_3_ salts (*Y* = H, halogen). In contrast to these known compounds, which are found in polar ortho­rhom­bic space groups, the structure reported here is in ortho­rhom­bic *Pbca*.

## Abstract 2

The structures of the iso­propyl­ammonium halidometallate salts (IPA^+^)_3_Co*X*_5_ (*X* = Cl, Br), (IPA^+^)_2_ZnCl_4_ and (IPA^+^)ZnCl_3_ are reported along with a survey of known iso­propyl­ammonium halidometallates, and a study of IPA^+^ cation geometry (both experimental and theoretical) juxtaposed with that of the trimethylammonium TMA^+^) cation.

## 1. Chemical context

The iso­propyl­ammonium cation (IPA^+^) is isomeric with the tri­methyl­ammonium (TMA^+^) cation, yet is less prominent as a counter-ion in metal halide structural chemistry. A search of the Cambridge Structural Database (CSD version 5.46; Groom *et al.*, 2016 ▸) yields only 28 hits with 19 different compounds for IPA^+^ metal halide salts in which at least three halide ions are bound to the metal with any type of bond and curated for strictly metal halides. A similar search for TMA^+^ salts yields 87 hits with 41 compounds. While the ions are isomeric, they can play distinctly different roles in metal halide structures. As a tertiary ammonium cation, TMA^+^ is a single hydrogen-bond donor, in contrast to the primary IPA^+^ cation. Meanwhile, the bulkiness of the iso­propyl­ammonium cation may template halidometallate structures different than those of other primary ammonium cations. For example, the *A*_2_*MX*′_4_ (*X*′ = halogen) layered perovskite structures that are commonly found with primary ammonium cations (Yu *et al.*, 2021 ▸), are only reported for large metal cation (Sn^2+^ or Pb^2+^) bromides or iodides (*vide infra*) with IPA^+^. The bulkiness of the IPA^+^ cation also breaks the layered perovskite structure into ribbons three complexes wide in the ambient temperature structure of (IPA^+^)_2_CuCl_4_ (CSD refcode: IPRACU; Anderson & Willett, 1974 ▸). This structure transforms at higher temperature in a thermochromic (green-to-yellow) phase transition to a structure consisting of isolated flattened CuCl_4_^2−^ tetra­hedra. (IPRACU01; Bloomquist, Willett & Dodgen, 1981 ▸). Given the unique features of the IPA^+^ cation, we have initiated a more thorough exploration of the IPA^+^ halidometallate structural space. We start by reporting structures of the new compounds (IPA^+^)_3_Co*X*_4_*X* (*X* = Cl, Br, compounds **I** and **II**, respectively), (IPA^+^)_2_ZnCl_4_, (compound **III**) and (IPA^+^)ZnCl_3_ (compound **IV**), a survey of known iso­propyl­ammonium halidometallates, and a study of IPA^+^ cation geometry (both experimental and theoretical) juxtaposed with that of TMA^+^.
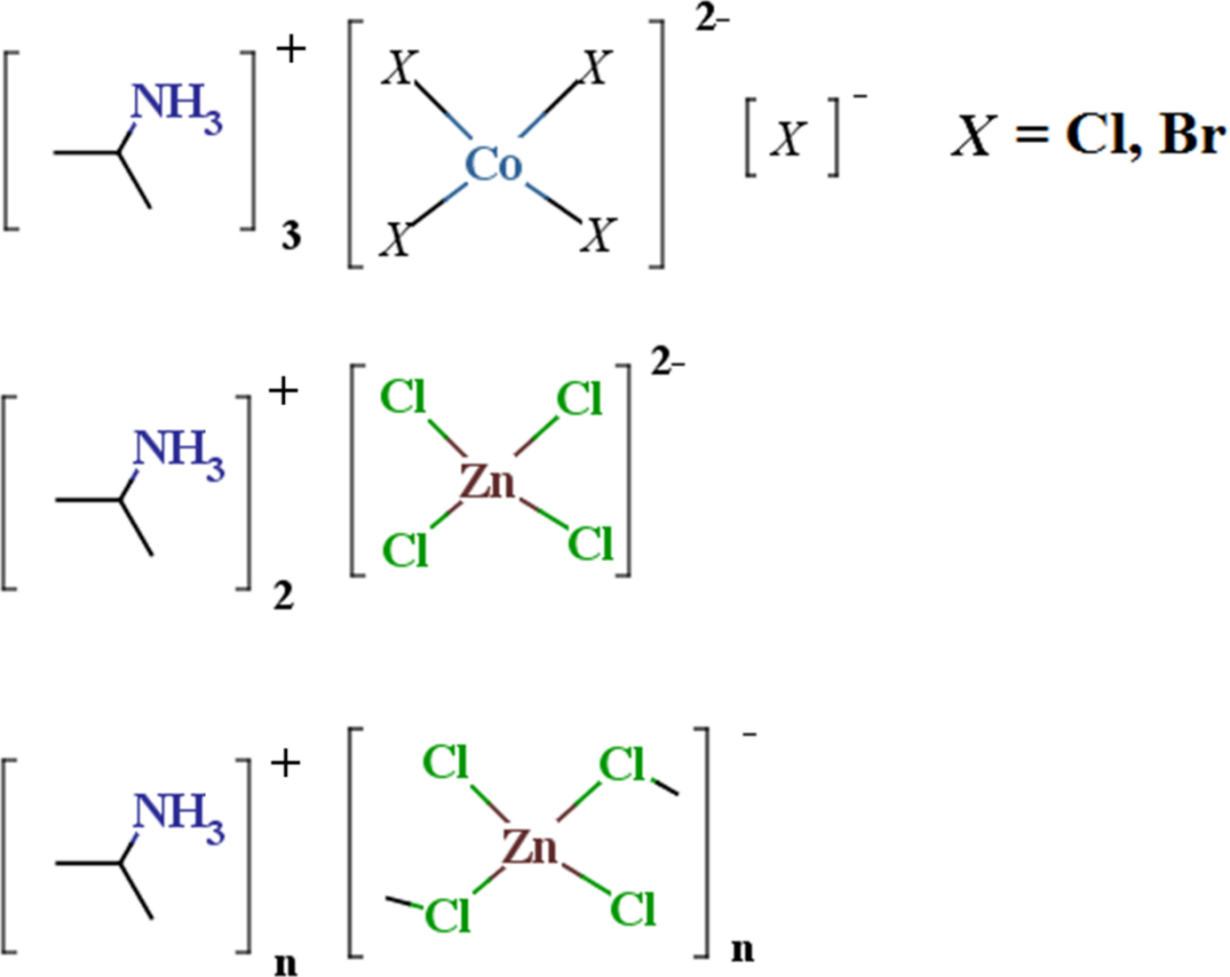


## 2. Structural commentary

### 2.1. (IPA^+^)_3_Co*X*_4_·*X*

The structures of the chloride and bromide salts are isomorphous. Blue crystals selected from samples grown from acidic aqueous solution with starting stoichiometries of 2:1, 1:1, and 1:2 in (IPA^+^)*X*:Co*X*_2_ are all found in this structure. Crystal growth from more Co*X*_2_-rich stoichiometries yields blue crystals inter­mixed with an increasing larger fraction of magenta crystals of Co*X*_2_·6H_2_O.

**Figure 1:**
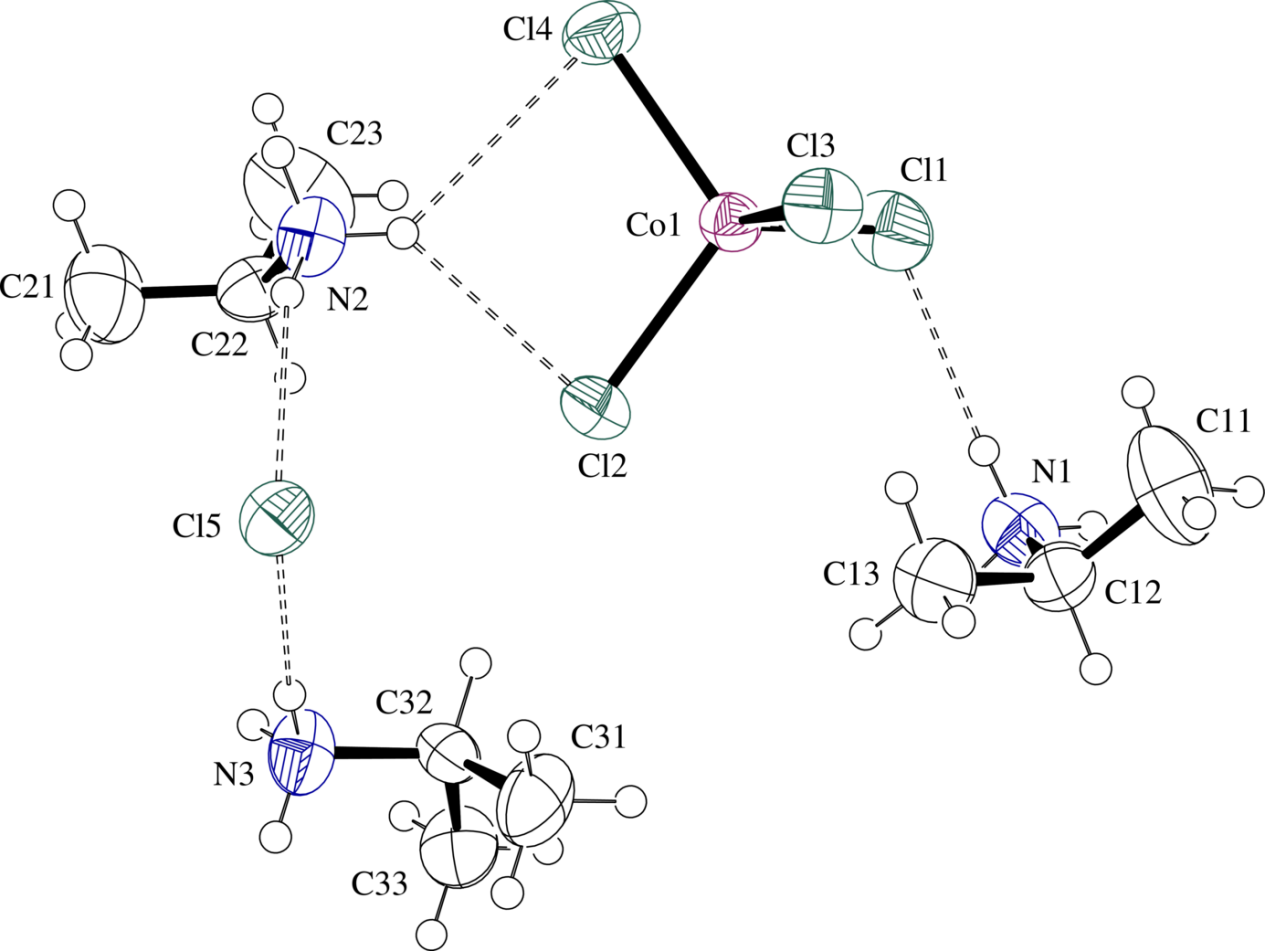
Displacement ellipsoid plot at the 50% level of the formula unit in (IPA^+^)_3_CoCl_4_·Cl (**I**) with labels for non-H atoms. Minor disorder components are omitted for clarity and H atoms are drawn as circles of arbitrary radii. N—H⋯Cl hydrogen-bonding inter­actions are drawn as dashed lines.

The structures consist of isolated Co*X*_4_^2−^ tetra­hedra surrounded by isolated halide and IPA^+^ ions. The complexes exhibit minor distortions from the expected tetra­hedral geometry. Co—*X* bond lengths are in the range 2.2561 (6)–2.2820 (6) Å for **I** and 2.3894 (7)–2.4110 (7) Å for **II** [average = 2.269 (11) Å for **I** and 2.410 (10) Å for **II**], while *X*—Co—*X* angles are in the range 104.72 (2)–112.61 (3)° for **I** and 105.72 (3)–112.63 (3)° for **II** [average = 109 (3)° for both **I** and **II**]. IPA^+^ cation #1 (N1) is ordered, while cation number #2 (N2) is slightly disordered in both structures [SOF (site occupation factor) = 0.851 (11) and 0.74 (3) for the major component in **I** and **II**, respectively]. The disorder corresponds to an ‘umbrella’ inversion relationship between the two components about N2. ‘Umbrella’ inversion disorder is also present for cation #3 (N3) in **I** only [SOF = 0.765 (5) for the major component]. Here N3 is found in different positions with two of the ammonium protons forming hydrogen bonds to the same acceptors in each component, but the third proton forming a hydrogen bond to a different acceptor. For **I** and **II**, respectively, Tables 1 ▸ and 2 ▸ contain selected geometric parameters and Figs. 1 ▸ and 2 ▸ present displacement ellipsoid plots with labels for non-H atoms of the formula units.

### 2.2. (IPA^+^)_2_ZnCl_4_

**Figure 3:**
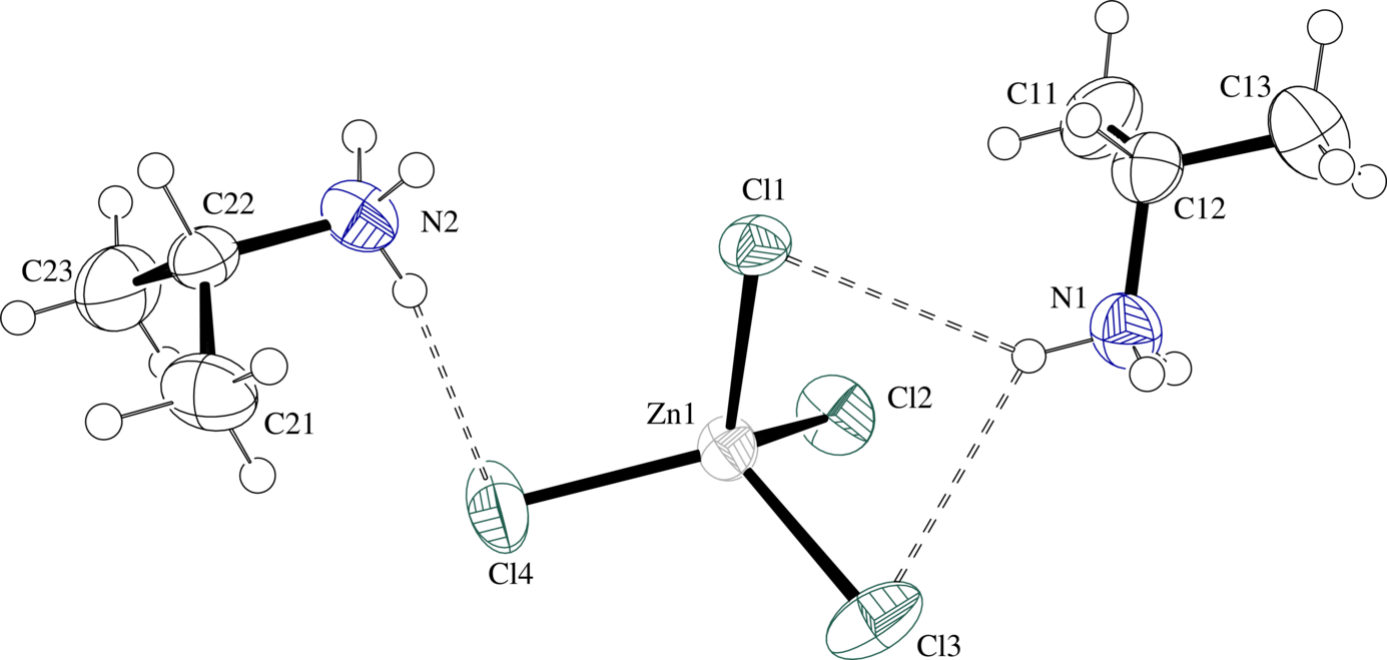
Displacement ellipsoid plot at the 50% level of the formula unit in (IPA^+^)_2_ZnCl_4_ (**III**) with labels for non-H atoms. H atoms are drawn as circles of arbitrary radii. N—H⋯Cl hydrogen-bonding inter­actions are drawn as dashed lines.

The most remarkable feature in this structure is the lack of disorder in the two symmetrically inequivalent IPA^+^ cations. The ZnCl_4_^2−^ complex is approximately tetra­hedral with Zn—Cl bond lengths ranging from 2.2346 (10)–2.2960 (8) Å [average = 2.27 (2) Å] and Cl—Zn—Cl angles ranging from 103.25 (4)–116.47 (4)° [average = 109 (5)°] with deviations from the ideal tetra­hedral values likely arising from hydrogen bonding inter­actions with the organic cations. Table 3 ▸ presents selected geometric parameters and Fig. 3 ▸ presents a displacement ellipsoid plot with labels for non-H atoms of the formula unit.

### 2.3. (IPA^+^)ZnCl_3_

**Figure 4:**
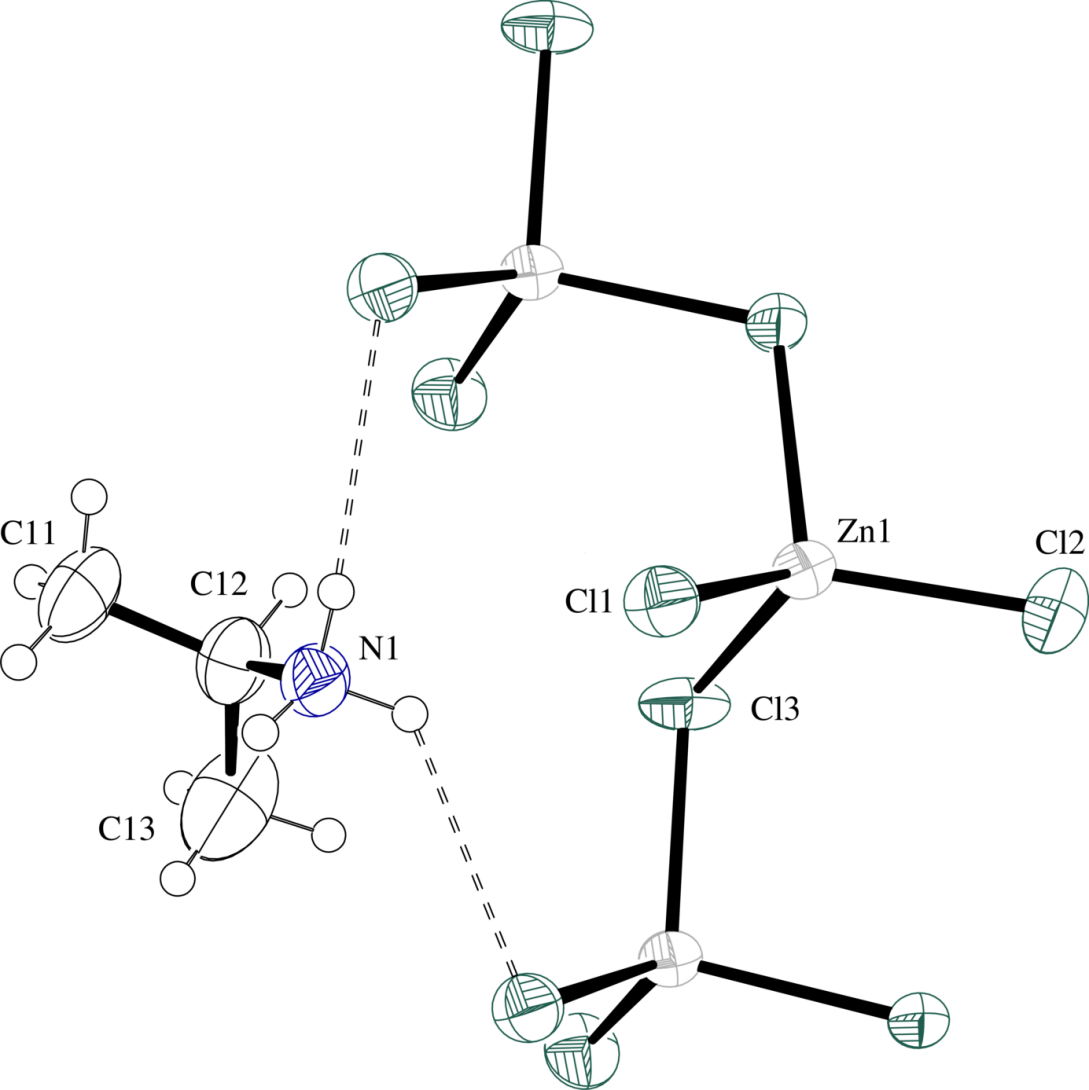
Displacement ellipsoid plot at the 50% level for the IPA^+^ cation and a section of the inorganic chain in (IPA^+^)ZnCl_3_ (**IV**) with labels for symmetry unique non-H atoms. H atoms are drawn as circles of arbitrary radii. N—H⋯Cl hydrogen-bonding inter­actions are drawn as dashed lines.

The structure consists of chains of corner-sharing ZnCl_4_ tetra­hedra separated by IPA^+^ ions. (ZnCl_3_^−^)_*n*_ chains propagate parallel to *a* with neighboring Zn^2+^ centers and bridging Cl^−^ ligands staggered about the *a*-glide plane. Weak electrostatic attraction between a bridging Cl atom and the next nearest neighboring Zn^2+^ center [Zn1⋯Cl3^ii^ = 4.1620 (7) Å; symmetry code: (ii) *x* − 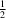, *y*, −*z* + 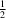] causes compression of the chain along the central axis to produce an inter­ior Cl—Zn—Cl angle less than 109.5°. Lateral repulsion between neighboring Zn^2+^ centers [Zn1⋯Zn1^i^ = 3.8557 (5) Å; symmetry code: (i) *x* + 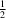, *y*, −*z* + 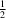) causes bulging of the chain and produces a bridging Zn—Cl—Zn angle larger than 109.5°. Bridging Zn—Cl bond lengths average 2.328 (14) Å, and are ∼0.1 Å longer than the average terminal Zn—Cl bond length [2.22 (2) Å]. The terminal Zn1—Cl1 bond is almost parallel (or anti­parallel) to *b*. Table 4 ▸ presents selected geometric parameters while Fig. 4 ▸ presents a displacement ellipsoid plot of the organic cation and a section of the inorganic chain with labels for symmetry unique non-H atoms.

### 2.4. Iso­propyl­ammonium and tri­methyl­ammonium cation geometry

A study of bond lengths and angles for both IPA^+^ and TMA^+^ cations in the CSD (Macrae *et al.*, 2020 ▸) was conducted with results summarized in the top section of Table 5 ▸. As pyramidal mol­ecular cations, both IPA^+^ and TMA^+^ are susceptible to twofold ‘umbrella’ inversion disorder. A cursory examination of disordered TMA^+^ cations indicates that twofold rotational disorder of the methyl groups about the N—H bond is another significant source of disorder not found with IPA^+^. Both cations show similar fractions of disordered structures (41/129 hits for IPA^+^, 96/318 hits for TMA^+^). The study was conducted for all structures, and for only non-disordered structures. Values for both groups were the same with minor improvements in variance about mean values for the non-disordered structures. For both cations, mean C—N bond lengths are smaller than the mean C—C length in IPA^+^, while the C—C—C angle in IPA^+^ shows the largest deviation from the ideal tetra­hedral. Histograms of these geometric parameters have been placed in the supporting information.

For the group of structures here, bond lengths and angles of the IPA^+^ cation correlate well with mean values for IPA^+^ obtained from the CSD. C—N bonds range from 1.477 (6)–1.502 (5) Å with a mean of 1.492 (8) Å. C—C bonds range from 1.443 (7)–1.527 (4) Å with a mean value of 1.502 (9) Å. The large C—C—C angle in IPA^+^ is well represented in these structures with a range of 110.8–114.9° and a mean value of 113.1 (13)°. C—C—N angles range from 106.6 (8)–113.2 (5)° with a mean value at the tetra­hedral ideal of 109.5 (14)°. (Minor components of disorder were not included in these ranges or mean values.)

DFT optimizations [B3LYP, 6311+G(d,p); *GAMESS* (Schmidt *et al.*, 1993 ▸)] of IPA^+^ and TMA^+^ cations *in vacuo* and in uniform dielectric (ɛ= 78.4) were performed with optimized geometric parameters presented in the lower section of Table 5 ▸. Calculated C—C and C—N bond lengths are slightly longer than experimental mean values, but could be accounted for by apparent bond-length foreshortening due to thermal motion in the database values. Calculated angles correlate well with database values, most notably in the large C—C—C angle for the IPA^+^ cation. A glaring exception is the C—N length in IPA^+^, which is ∼0.03 Å *longer* than the C—C distance *in vacuo*. However, this distance reduces substanti­ally in dielectric where it is slightly less than the C—C distance. MOL files from the optimizations have been placed in the supporting information.

## 3. Supra­molecular features

### 3.1. (IPA^+^)_3_Co*X*_4_·*X*

**Figure 5:**
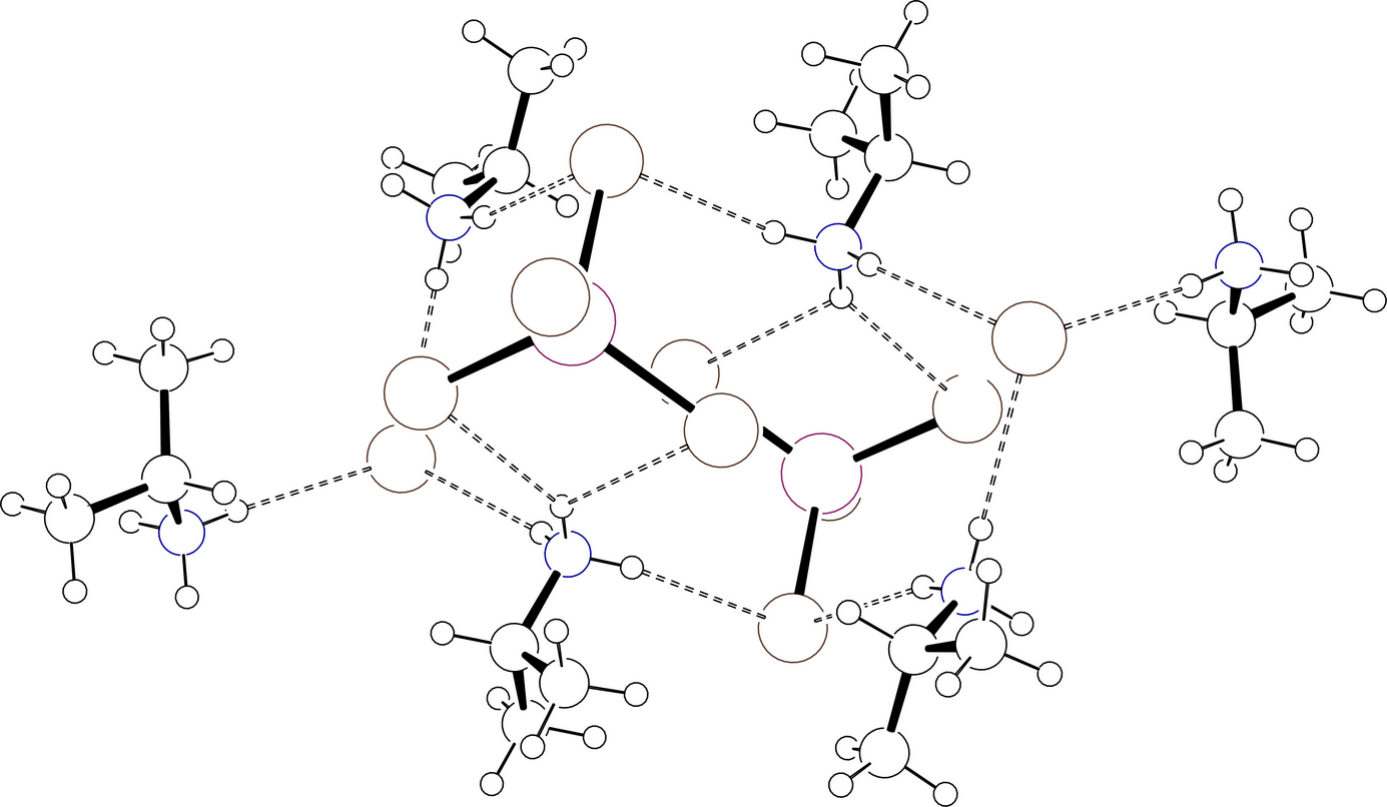
Plot of the hydrogen bonded cluster in (IPA^+^)_3_CoBr_4_·Br consisting of a pair of CoBr_4_^2−^ complexes and a pair of uncoordinated Br^−^ ions. Atoms are drawn as circles of arbitrary radii, hydrogen bonds are drawn as dashed lines, and minor disorder components are omitted for clarity.

**Figure 6:**
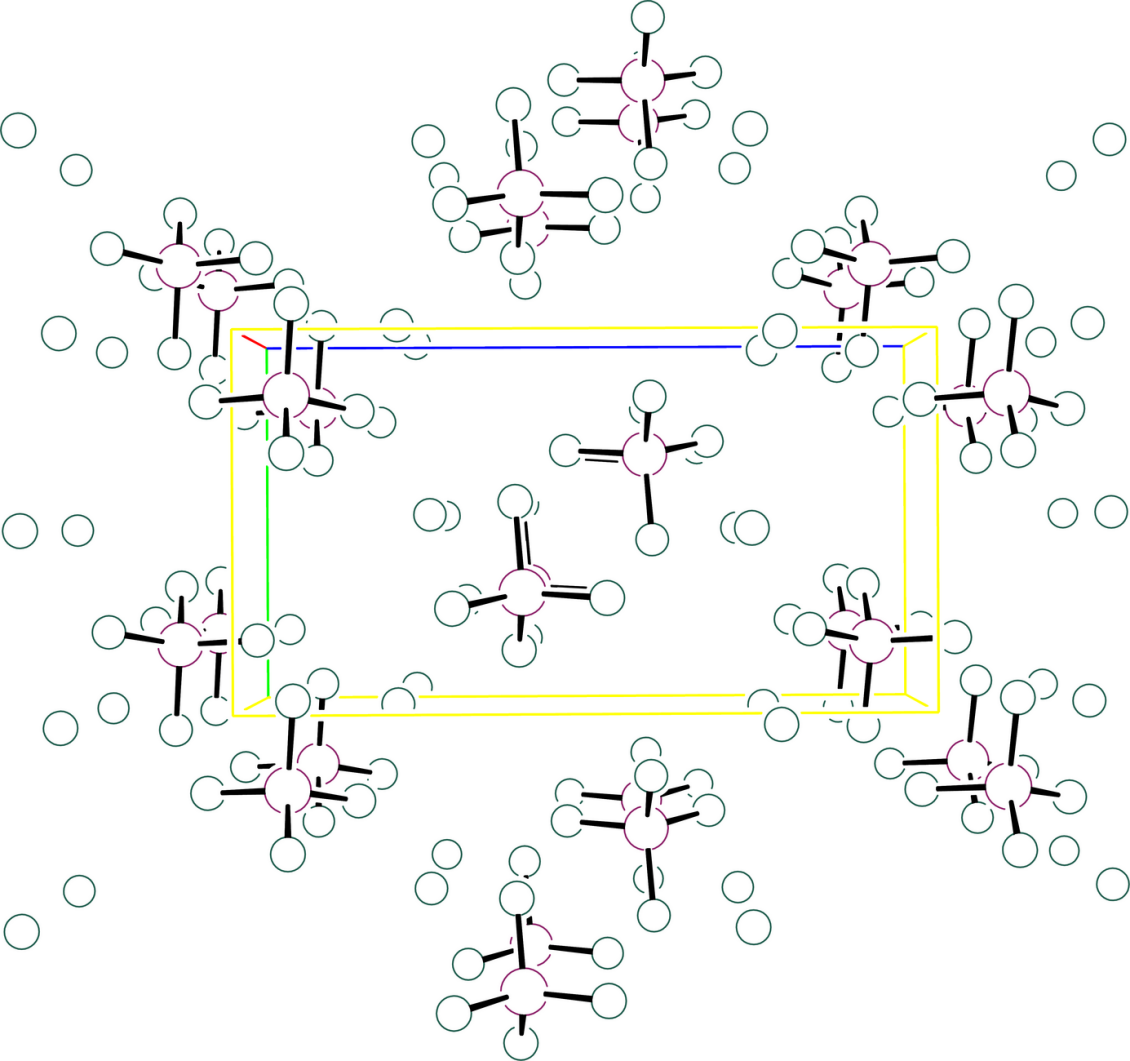
Packing diagram showing the clusters in (IPA^+^)_3_CoCl_4_·Cl stacked along *a*. The *b* axis is vertical and the *c* axis horizontal. Atoms are drawn as circles of arbitrary radii and organic cations are omitted for clarity.

**Figure 7:**
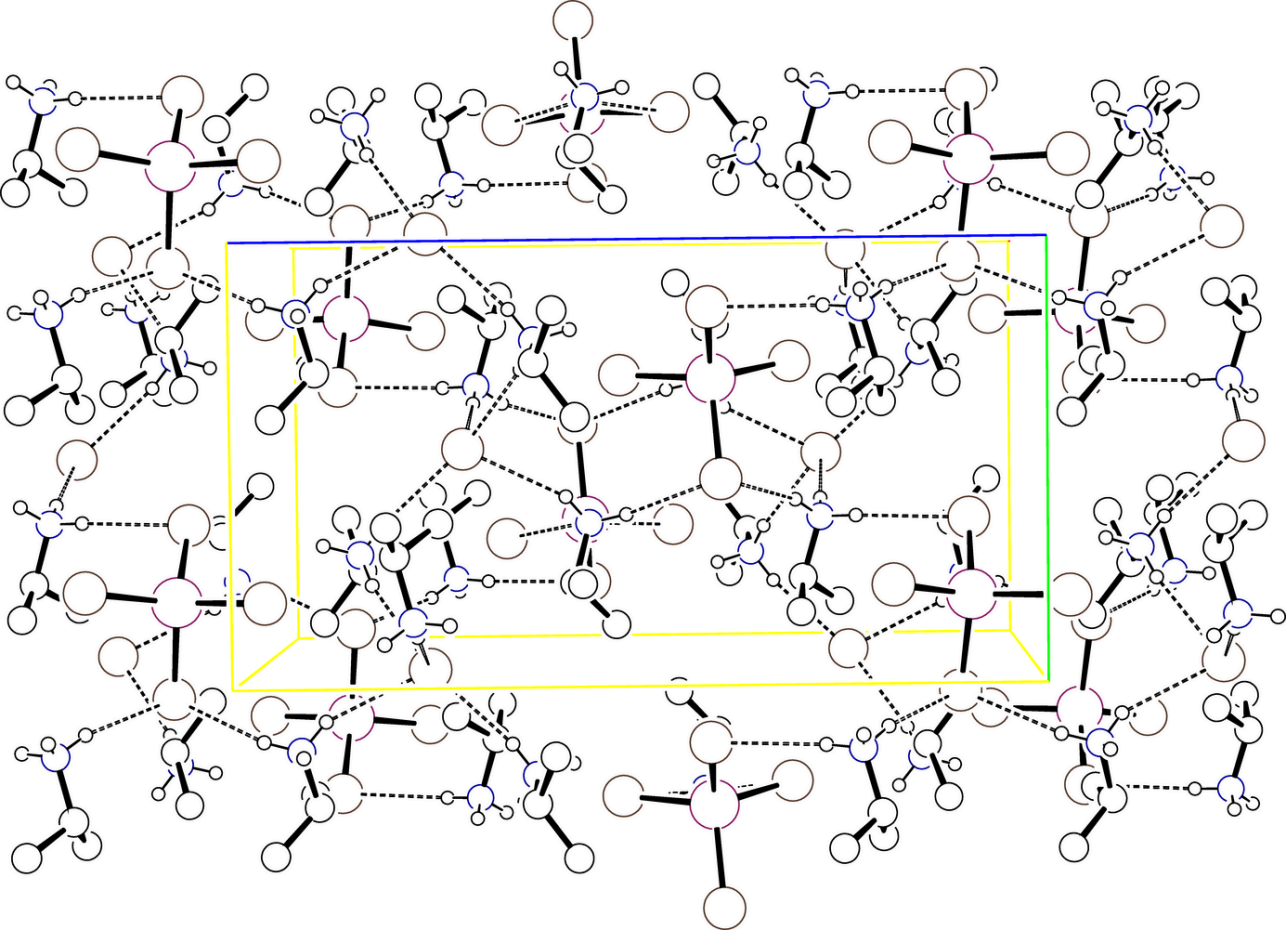
Packing diagram for (IPA^+^)_3_CoBr_4_·Br in a similar orientation as in Fig. 6 ▸ but including the organic cations to depict the extensive hydrogen-bonding network. Atoms are drawn as circles of arbitrary radii. H atoms bound to C and minor disorder components are omitted for clarity. Hydrogen bonds are drawn as dashed lines.

IPA^+^ cation #2 forms hydrogen bonds to two neighboring complexes and to two isolated halide ions that groups pairs of complexes and isolated halide ions into clusters about an inversion center. The cluster axis, as defined by the intra­cluster Co⋯Co vector, is highly canted relative to each of the unit cell axes forming angles of 43.83(<1)° with respect to *a*, 58.74(<1)° with respect to *b*, and 63.81(<1)° with respect to *c* for **I** [44.14(<1)°, 58.51(<1)°, and 64.49(<1)°, respectively, for **II**]. The clusters stack along *a* with each stack surrounded by six neighboring stacks. The other two IPA^+^ cations use one or two protons to form hydrogen-bonding contacts with halides in a given cluster while the remaining protons form contacts with clusters in neighboring stacks. In this way, the stacks are woven together with an extensive hydrogen-bonding network. Of note are methine C—H⋯*X* contacts from cations #1 and #3 (major component only) in the range 2.81–3.06 Å (and with C—H⋯*X* angles > 140°) to bound halide ions. These are significantly shorter and more direct than those found for compound **III**, where all contacts are greater than 3.16 Å with C—H⋯*X* angles < 115°. The weak C—H hydrogen-bonding inter­actions in **I** and **II** thus might be responsible for templating a different structure. Fig. 5 ▸ presents a plot of the cluster of hydrogen-bonded pairs in **II**, Fig. 6 ▸ presents a unit-cell packing diagram with organic cations absent for **I** and Fig. 7 ▸ presents a unit-cell packing diagram with the organic cations present that depicts the hydrogen-bonding network in **II**. Tables 6 ▸ and 7 ▸ present selected hydrogen-bonding parameters for **I** and **II**, respectively.

### 3.2. (IPA^+^)_2_ZnCl_4_

**Figure 8:**
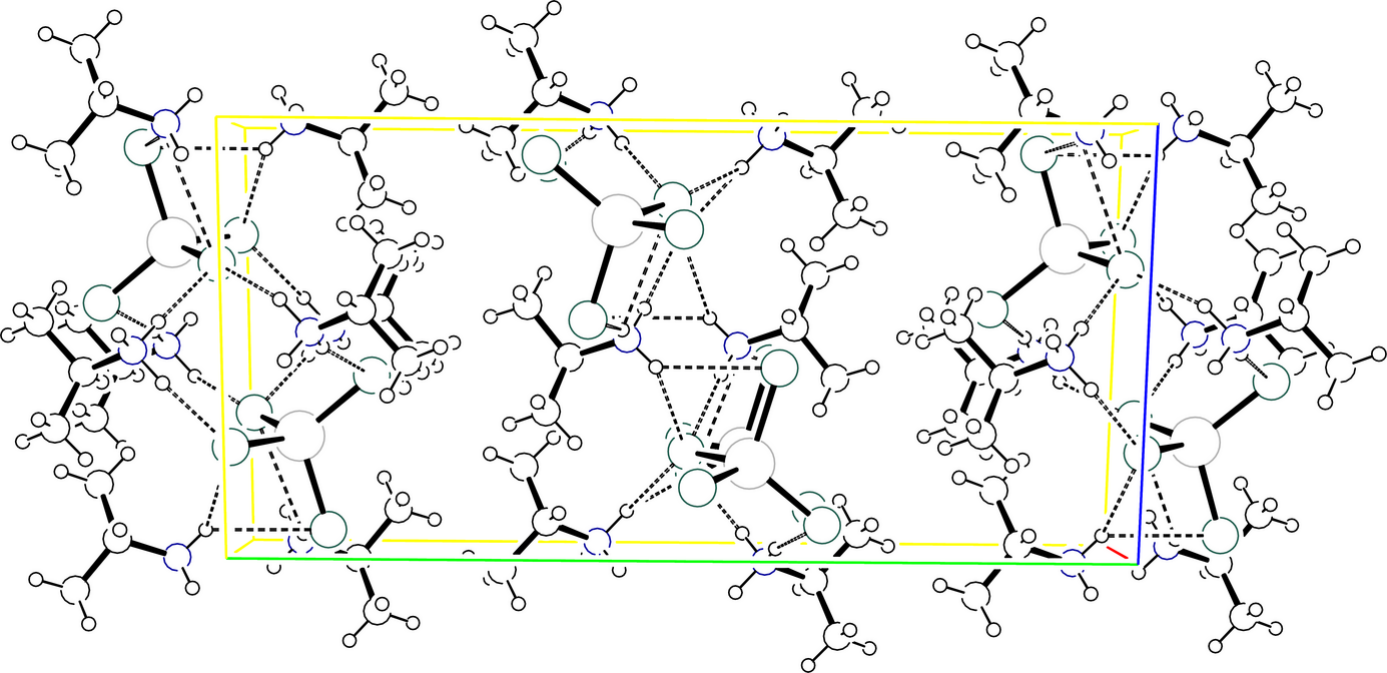
Packing diagram for (IPA^+^)_2_ZnCl_4_ viewed down *a* with the *b* axis horizontal and the *c* axis vertical. Atoms are drawn as circles of arbitrary radii, H atoms bound to C are omitted for clarity, and hydrogen bonds are drawn as dashed lines.

The structure contains double layers of ZnCl_4_^2−^ tetra­hedra parallel to [010]. Metal complexes in a given layer are related to one another by translations along *a* or *c*. Complexes in the companion layer are offset with the two layers related by inversion. The ammonium headgroups of the IPA^+^ cations are deeply embedded within the double layer, and form multiple hydrogen bonds to chloride ligands in both layers to tie the complexes together. The methine C—H bond is approximately parallel to the layer with the methyl groups directed outward to abut the methyl groups projecting from the neighboring double layer. Symmetry-unique IPA^+^ cations are found on both sides of the double layer, but with their ammonium head groups tilted in different directions. Fig. 8 ▸ presents a unit-cell packing diagram while Table 8 ▸ presents selected hydrogen-bonding parameters.

### 3.3. (IPA^+^)ZnCl_3_

**Figure 9:**
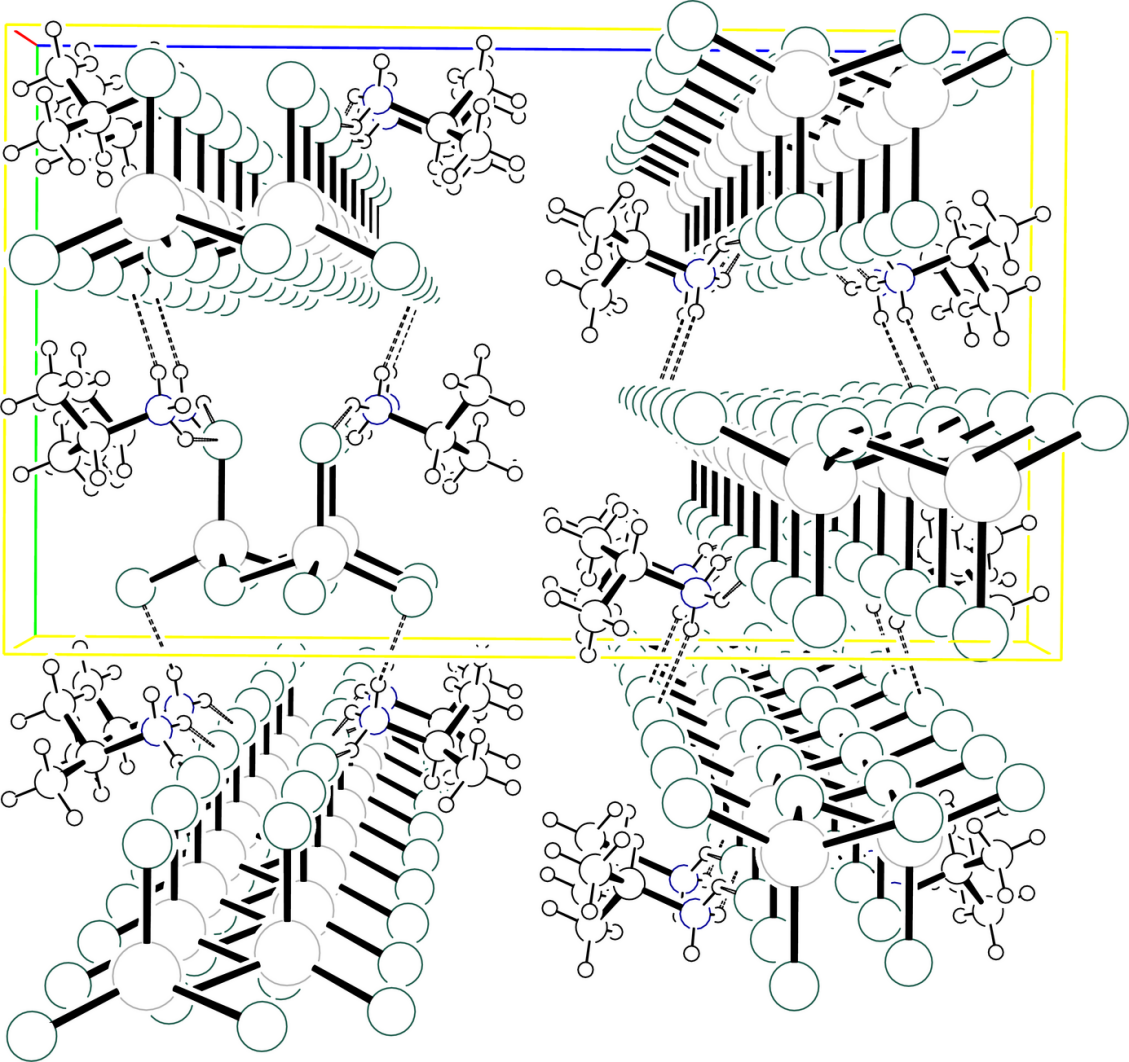
Packing diagram for (IPA^+^)ZnCl_3_ viewed along *a* with the *b* axis vertical and the *c* axis horizontal. Atoms are drawn as circles of arbitrary radii and hydrogen bonds are drawn as dashed lines.

All protons of the ammonium headgroup are involved in hydrogen bonding to terminal chloride ligands. Two protons form hydrogen bonds (one strong and one weak) to terminal ligands (Cl1) attached to next nearest neighbor Zn^2+^ centers (on the same side of the glide plane) while the third forms the shortest hydrogen bond to a terminal ligand (Cl2) on a neighboring chain. This links chains with the same direction for their Zn1—Cl1 bonds into slabs in the *ab* plane. The slabs are stacked along *c* with neighboring slabs related by inversion symmetry to reverse the direction of the Zn1—Cl1 bond. The isopropyl groups project away from the slab to abut isopropyl groups from a neighboring slab. Of note is a direct methine C—H⋯Cl2 inter­action < 3.0 Å in length. Fig. 9 ▸ presents a unit-cell packing diagram while Table 9 ▸ presents selected hydrogen-bonding parameters.

## 4. Database survey

### 4.1. Iso­propyl­ammonium halidometallates

The only structure containing isolated tetra­halidometallate complexes reported in the Cambridge Structural Database is the classic thermochromic compound (IPA^+^)_2_CuCl_4_ (*vide supra*). (IPA^+^)_2_SbBr_5_ has been reported as occurring with an isolated five-coordinate metal complex (QQQGOS), although without 3D coordinates (Jha & Rizvi, 1974 ▸). Crystallographic information on several other salts of IPA^+^ with isolated metal complexes have been reported. The most pertinent is the mixed valence compound (IPA^+^)_8_(SbCl_6_)^−^(SbCl_6_)^3−^·4Cl^−^ with isolated chloride ions (IPASBC; Birke *et al.*, 1976 ▸). (IPA^+^)_2_ReCl_6_ is known (JASHAM; Bettinelli *et al.*, 1989 ▸) with reports of Pt and Sn analogs (QQQGBP and QQQGBS, respectively; Bhalla & Cross, 1974 ▸ – 3D coordinates not reported). The Re salt exhibits a layered arrangement of metal complexes sandwiched by bilayers of IPA^+^ cations.

(IPA^+^)_3_SnI_5_, -PbBr_5_, and -PbI_5_ are stoichiometrically identical to the title cobalt compounds, but these structures consist of zigzag chains of corner-sharing octa­hedra that result from dimensional breakdown of the 2D layered perovskite network [JANSEZ (Stoumpos *et al.*, 2017 ▸), ROLWUO (Ru *et al.*, 2023 ▸), and TOSSEC (Hartono *et al.*, 2019 ▸)]. (IPA^+^)_3_Sn_2_I_7_ consists of a double-layered perovskite structure with one IPA^+^ cation located in the perovskite cavity and three different phases observed (JAMWIG, JAMWIG01, JAMWIG02; Stoumpos *et al.*, 2017 ▸). The bulky IPA^+^ cation in the perovskite cavity renders this compound metastable. The mixed organic cation salts (IPA^+^)_2_(Tz^+^)_*n*–1_Pb_*n*_Br_3*n*+1_ (Tz = 1,2,4-triazolium) provide a range of perovskite structure types (Li *et al.*, 2022 ▸) with a single layered perovskite structure for *n* = 1 (FIPDUH), and two- and three-layer perovskites with the planar Tz cation occupying the perovskite cavity (FINVAD and FINVEH, respectively). A subsequent study of FIPDUH (He *et al.*, 2024 ▸) reveals that the ambient temperature phase in polar monoclinic *Cc* (FIPDUH01) transforms at 340.5 K to a phase found in centrosymmetric ortho­rhom­bic *Pbca* (FIPDUH02). In turn, this phase transforms at 370.5 K to a phase found in ortho­rhom­bic *C*m*ca* (FIPDUH03).

(IPA^+^)CuCl_3_ (Roberts *et al.*, 1981 ▸) is a thermochromic compound in which the low-temperature phase (brown) consists of stacks of Cu_2_Cl_6_^2−^ quasi-planar complexes (IPAMCU01). This transforms to a high-temperature phase (orange) consisting of chains of face-sharing CuCl_6_ octa­hedra separated by disordered IPA^+^ cations (IPAMCU). The bromide analog (IPBRCU; Bloomquist & Willett, 1981 ▸) is isomorphous. (IPA^+^)_4_Cd_3_Cl_10_ belongs to the Cs_4_Mg_3_F_10_ family of layered structures consisting of trinuclear sections of face-sharing CdCl_6_ octa­hedra linked together in a zigzag 2D network (Gagor *et al.*, 2011 ▸). Here three different phases are observed with varying degrees of disorder of the IPA^+^ cation (IPEMAS, IPEMAS01, and IPEMAS02). Parallel linear chains of face-sharing CdBr_6_ octa­hedra are found for (IPA^+^)CdBr_3_ (WIZSON; Ishihara *et al.*, 1999 ▸) and -PbI_3_ (Fedoruk-Piskorska *et al.*, 2024 ▸) in a hexa­gonal perovskite arrangement, and likewise for (IPA^+^)GeI_3_ (YUJYUZ; Stoumpos *et al.*, 2015 ▸), albeit with half the chains perpendicular to the other half. The PbI_3_ salt transforms from polar monoclinic *P*2_1_ (COYBOL) to centrosymmetric ortho­rhom­bic *C*m*c*m (COYBOL01) above 284.1 K.

### 4.2. *A*_3_(*MX*′_4_)*X*′ and *B*(*MX*′_4_)*X*′

A search of the Cambridge Structural Database yields 24 distinct compounds (from 35 hits) that fit the *A*_3_(*MX*′_4_)*X*′ stoichiometry (where *A* = monopositive organic cation, *M* = any metal, and *X*′ = halogen). Six are found with *M* = Co and one found for *M* = Zn. *M* = Mn is most numerous with nine compounds, *M* = Cu next with five, and one each for *M* = Ni, Pd, Hg. For *M* = Pd, the organic cation is the isomeric TMA^+^ ion with square-planar PdCl_4_^2−^ (BIJNIR; Zveguintzoff *et al.*, 1981 ▸). The lone reported *M* = Zn compound is with *tert*-butyl­ammonium (WAZXEA; Ishida & Kashino, 1993 ▸). More numerous are *B*(*MX*′_4_)*X*′ compounds in which *B* is a tripositive cation: 52 compounds from 54 hits. Compounds in which *M* = Zn account for 21 of these and greatly outnumber the seven compounds with *M* = Co. Based on reported structures to date, it appears that Co^2+^ has the greater propensity to form a tetra­hedral complex with an uncoordinated halide if the cation is monopositive, whereas Zn^2+^ has the greater propensity with tripositive cations, which may account for the difference in structures here.

### 4.3. *A*_2_CoCl_4_*versus A*_2_ZnCl_4_

The tetra­hedral CoCl_4_^2−^ and ZnCl_4_^2−^ complexes are well known: a simple search in the Cambridge Structural Database yields 623 hits for CoCl_4_ and 1141 hits for ZnCl_4_ complexes. There are 54 instances in which both Co and Zn form an *A*_2_*M*Cl_4_ compound with the same monopositive organic cation – including one instance of a mixed-metal structure (BICDOK; Behrens *et al.*, 2022 ▸). The vast majority of Co/Zn pairs are isostructural, at least for one solid phase, with only three examples of completely dissimilar structures. This is not unexpected since the two metal ions have very similar radii [58 pm for Co^2+^ and 60 pm for Zn^2+^, Shannon effective ionic radii for tetra­hedral coordination (Shannon, 1976 ▸)]. For example, in the (1,3,4-tri­methyl­pyridinium)_2_*M*Cl_4_ series (Bond, 2009 ▸, 2019 ▸) those compounds with smaller ionic radii metal ions [*M* = Co (COQZAM), Ni (LOHPIK), Cu (QUBHIF), Zn (COMGAP)] are isostructural (ortho­rhom­bic *Fdd*2) with a crossover to monoclinic *C*2*/c* structures for larger radii metal ions [*M* = Mn (YOKNIY), Cd (LOGRIL)]. In particular, mol­ecular volumes for the Co and Zn members of this series agree within 0.07%. Of special note is the recently reported (2-chloro-*N*,*N*-di­methyl­ethanaminium)_2_ (Co or Zn)Cl_4_ system (VOTDUH or VOTFAP, respectively; McGinness *et al.*, 2024 ▸) in which the two structures are almost identical, right down to the level of disorder of the organic cation. A table containing *A*_2_CoCl_4_ and *A*_2_ZnCl_4_ compounds with common *A* cations is included in the supporting information.

### 4.4. *AMX*_3_ structures containing chains of corner-sharing tetra­hedra

Five *A*ZnCl_3_ compounds with chains of corner-sharing tetra­hedra are reported in the Cambridge Structural Database among eight hits, almost all of recent origin. The earliest report is for (tetra­methyl­ammonium)ZnCl_3_ (OGEYOP; Choi *et al.*, 2009 ▸). The remaining compounds are found with halogeno-substituted tetra­methyl­ammonium cations (WUKROM, WUKRUS, WUKSAZ, WUKSED; Chen *et al.*, 2020 ▸). In all cases these compounds are found in polar ortho­rhom­bic space groups, in contrast to the primary ammonium salt presented here. The chains in these compounds have a staggered arrangement similar to that in (IPA^+^)ZnCl_3_, although without formal glide-plane symmetry. In this case, however, bridging ligands are arranged in a line parallel to the chain axis while the terminal Zn—Cl bonds (comparable to Zn1—Cl1 in this structure) are twisted inward. These terminal ligands now make the long contact to the Zn^2+^ centers with distances ∼0.15 Å less than the contact distance with the bridging ligand in the IPA^+^ salt [distances range from 3.973–4.059 Å with an average of 4.03 (3) Å]. The shorter contact distance also reduces the Zn⋯Zn distance to an average of 3.758 (14) Å (range: 3.738–3.778 Å), bridging Zn—Cl—Zn angles less than 109.5°, and more acute inter­ior Cl—Zn—Cl angles. This reduction in angle brings the metal centers closer to the chain axis, which results in a lengthening of the chain to give an average chain repeat distance of 7.20 (3) Å compared to 6.3131 (3) Å in the IPA^+^ salt.

Similar chains with terminal ligands twisted inward are found for twelve *AMX*_3_ systems, six of which are tetra­methyl­ammonium salts of mercury(II) halides: -HgCl_3_ [BOPXIN(01, 02, 04); Rao & Rajaram, 1982 ▸; Sikirica *et al.*, 1982 ▸; Lambarki *et al.*, 2018 ▸], -HgBr_3_ [TMAHGB(01); White, 1963 ▸; Sikirica *et al.*, 1982 ▸], -HgI_3_ (TMAIHG11; Sharutin *et al.*, 2011 ▸), and mixed halide systems -HgCl_0.63_Br_2.37_, -HgBrI_2_, HgCl_0.45_I_2.55_ (OSOFAG, BORYIC04, TMAIHG12 respectively; Yang *et al.*, 2021 ▸). However, in these cases the long *M*⋯*X* contact to a neighboring terminal halide has become short enough to be considered a semi-coordinate bond with the structure perhaps better described as chains of asymmetrically bibridged trigonal bipyramids. Tri­iodo­cadmate(II) compounds are known with pyrrolidinium [IVIGUS(01); Rok *et al.*, 2021 ▸], ethyl­tri­methyl­ammonium, and TMA^+^ (KELNAS and KELNEW, respectively; Sharutin *et al.*, 2012 ▸). In (pyrrolidinium)CdI_3_, half the chains are perpendicular to the other half. All of these systems crystallize in polar space groups, while the remaining three chain structures are found in centrosymmetric monoclinic *P*2_1_/*c*: (tri­methyl­sulfonium)CdI_3_ [NUDZAN(01); Svensson *et al.*, 1998 ▸], (tri­methyl­ammonium)HgI_3_ (PIJRAB; Geselle *et al.*, 1993 ▸), and (4′,5′-bis­(methyl­sulfan­yl)- 4,5-(ethyl­enedi­thio)­tetra­thia­fulvalene) HgI_3_ (QOLGUU; Yang *et al.*, 2009 ▸). The chains in both PIJRAB and QOLGUU contain substanti­ally asymmetric monobridging.

(Chloro-(1,4,10,13-tetra­oxa-7,16-di­thia­cyclo­octa­deca­ne)HgCl_3_ (LUHDID; Kang *et al.*, 2012 ▸) contains chains with parallel terminal Hg—Cl bonds, as in (IPA^+^)ZnCl_3_, except the bridging ligands are not staggered. In (methyl­ammonium)HgI_3_ (DEBMEC; Korfer *et al.*, 1985 ▸), chains arrange terminal *M*—Cl bonds so they are splayed uniformly about the chain axis. Spiral chains are found in (di­methyl­ammonium)HgBr_3_ (QACFIJ; Terao *et al.*, 1998 ▸) and in (methyl­ammonium)HgCl_3_ (QQQBVJ21; Salah *et al.*, 1982 ▸) and -HgClBr_2_ (SOPDOQ; Hassen *et al.*, 1997 ▸).

## 5. Synthesis and crystallization

1.3 mL of neat iso­propyl­amine (*d* = 0.69 g mL^−1^, 15 mmol) were neutralized by slow, dropwise addition of concentrated HCl until the solution tested acidic with litmus paper. Distilled water was added to give a total volume of 70 mL. 40 mL, 20 mL and 10 mL of this solution were placed in different beakers with addition of distilled water, as needed, to give a total volume of 40 mL of solution in each. 1.0 g of CoCl_2_·6H_2_O (4.2 mmol) were added to each beaker with stirring until completely dissolved to give approximately 2:1, 1:1, and 1:2 stoichiometries, respectively, of organic cation: CoCl_2_. 40 mL of concentrated hydro­chloric acid were then added to each beaker and crystals then grown by slow evaporation. The resulting solid mass was gently pressed between tissues to wick away mother liquor, then stored in a screw cap vial. The same procedure was used in crystallizations with CoBr_2_·6H_2_O and ZnCl_2_·H_2_O except that 1.0 mL and 2.0 mL, respectively, of neat iso­propyl­amine were used.

## 6. Refinement

Crystal data, data collection, and structure refinement details are summarized in Table 10 ▸.

### 6.1. (IPA^+^)_3_Co*X*_4_·*X*

Non-H atoms were identified in the initial structure solution, except those of minor disorder components. Anisotropic refinement of all atoms yielded anomalously large displacement ellipsoids for some C and N atoms with suggestions by the *SHELX* software to split these into disordered pairs. In addition, difference map peaks were found close to methine C atoms indicating ‘umbrella’ inversion disorder. A twofold disordered model for these organic cations was pursued with each pair refined constrained so that the sum of their site occupation factors equal 1.0. H atoms were constrained to an idealized geometry and refined in a riding model with displacement parameters equal to 1.5×*U*_iso_ of the parent atom.

### 6.2. (IPA^+^)_2_ZnCl_4_ and (IPA^+^)ZnCl_3_

All non-H atoms were identified from the initial structure solution and were subjected to anisotropic refinement. H atoms were visible in subsequent electron-density difference maps and were refined to reasonable geometries and displacement parameters for those bound to N. Those bound to C were constrained to an idealized geometry and refined in a riding model with displacement parameters fixed at 1.5×*U*_iso_ of the parent C-atom.

## Supplementary Material

Crystal structure: contains datablock(s) global, I, II, III, IV. DOI: 10.1107/S2056989025006103/nu2010sup1.cif

Structure factors: contains datablock(s) I. DOI: 10.1107/S2056989025006103/nu2010Isup2.hkl

Structure factors: contains datablock(s) II. DOI: 10.1107/S2056989025006103/nu2010IIsup3.hkl

Structure factors: contains datablock(s) III. DOI: 10.1107/S2056989025006103/nu2010IIIsup4.hkl

Structure factors: contains datablock(s) IV. DOI: 10.1107/S2056989025006103/nu2010IVsup5.hkl

Histograms of geometric parameters for IPA+ and TMA+ cations obtained from the Cambridge Structural Database. DOI: 10.1107/S2056989025006103/nu2010sup6.docx

Table of A2CoCl4 and A2ZnCl4 compounds which share a common A cation. DOI: 10.1107/S2056989025006103/nu2010sup7.docx

MOL file for optimized geometry of the IPA+ cation in vacuo. DOI: 10.1107/S2056989025006103/nu2010sup8.mol

MOL file for optimized geometry of the IPA+ cation in uniform dielectric. DOI: 10.1107/S2056989025006103/nu2010sup9.mol

MOL file for optimized geometry of the TMA+ cation in vacuo. DOI: 10.1107/S2056989025006103/nu2010sup10.mol

MOL file for optimized geometry of TMA+ cation in uniform dielectric. DOI: 10.1107/S2056989025006103/nu2010sup11.mol

CCDC references: 2471094, 2471093, 2471092, 2471091

Additional supporting information:  crystallographic information; 3D view; checkCIF report

## Figures and Tables

**Figure 2:**
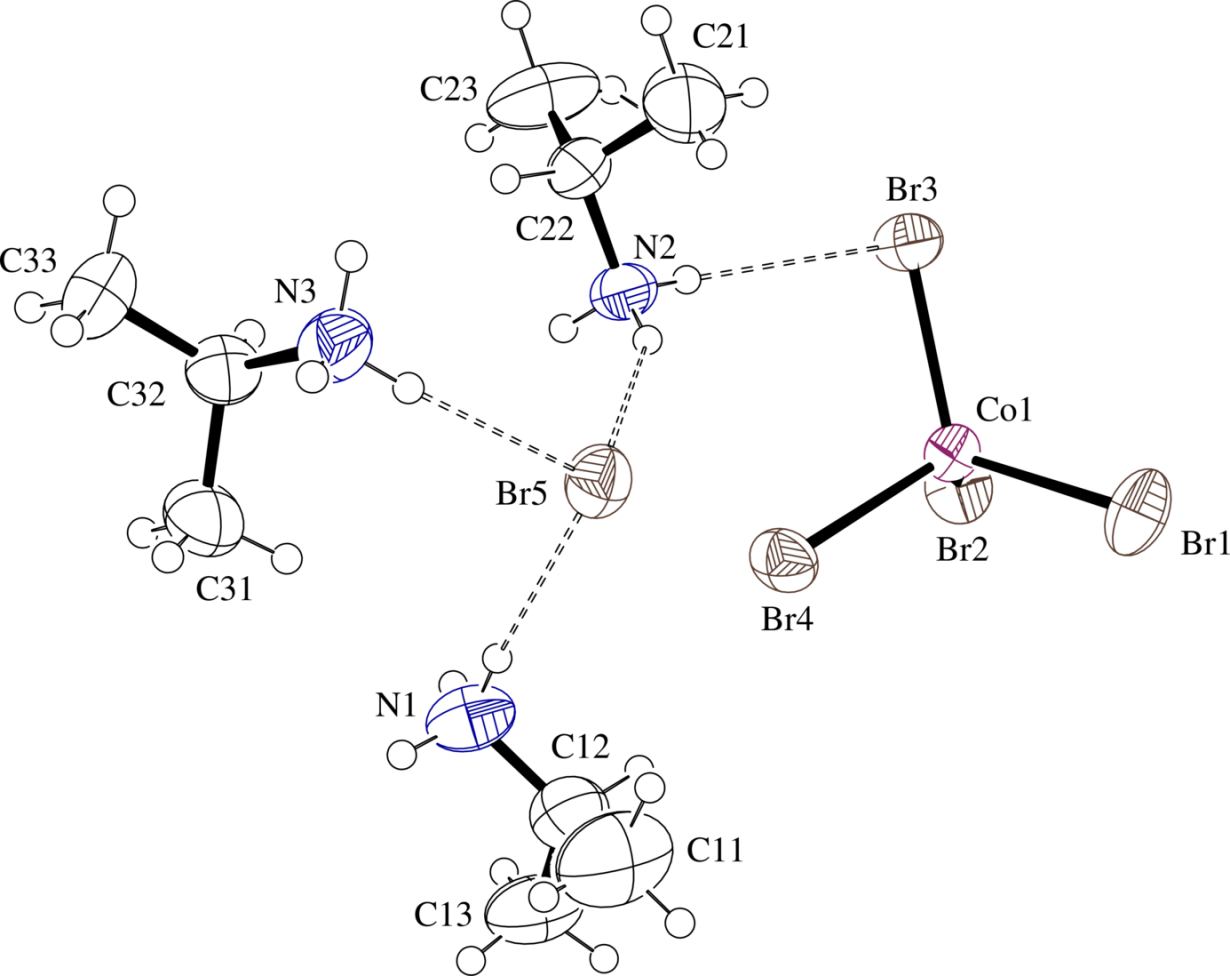
Displacement ellipsoid plot at the 50% level of the formula unit in (IPA^+^)_3_CoBr_4_·Br (**II**) with labels for non-H atoms. Minor disorder components are omitted for clarity and H atoms are drawn as circles of arbitrary radii. N—H⋯Br hydrogen-bonding inter­actions are drawn as dashed lines.

**Table 1:** Selected geometric parameters (Å, °) for **I**[Chem scheme1]

Co1—Cl1	2.2561 (6)	Co1—Cl3	2.2820 (6)
Co1—Cl2	2.2600 (6)	Co1—Cl4	2.2762 (6)
			
Cl1—Co1—Cl2	112.61 (3)	Cl2—Co1—Cl3	106.68 (3)
Cl1—Co1—Cl3	111.87 (3)	Cl2—Co1—Cl4	109.22 (3)
Cl1—Co1—Cl4	111.34 (3)	Cl3—Co1—Cl4	104.72 (2)

**Table 2:** Selected geometric parameters (Å, °) for **II**[Chem scheme1]

Co1—Br1	2.3939 (7)	Co1—Br3	2.4102 (7)
Co1—Br2	2.3894 (7)	Co1—Br4	2.4110 (7)
			
Br1—Co1—Br2	112.63 (3)	Br2—Co1—Br3	106.91 (3)
Br1—Co1—Br3	112.09 (3)	Br2—Co1—Br4	110.57 (3)
Br1—Co1—Br4	108.70 (3)	Br3—Co1—Br4	105.72 (3)

**Table 3:** Selected geometric parameters (Å, °) for **III**[Chem scheme1]

Zn1—Cl1	2.2960 (8)	Zn1—Cl3	2.2828 (10)
Zn1—Cl2	2.2681 (9)	Zn1—Cl4	2.2347 (10)
			
Cl1—Zn1—Cl2	110.35 (4)	Cl2—Zn1—Cl3	101.57 (4)
Cl1—Zn1—Cl3	103.26 (4)	Cl2—Zn1—Cl4	116.46 (4)
Cl1—Zn1—Cl4	109.44 (4)	Cl3—Zn1—Cl4	114.81 (5)

**Table 4:** Selected geometric parameters (Å, °) for **IV**[Chem scheme1]

Zn1—Cl1	2.2394 (7)	Zn1—Cl3	2.3182 (7)
Zn1—Cl2	2.2089 (7)	Zn1—Cl3^i^	2.3387 (7)
			
Cl1—Zn1—Cl2	113.89 (3)	Cl3—Zn1—Cl3^i^	102.19 (2)
Cl1—Zn1—Cl3	106.89 (3)	Zn1—Cl3—Zn1^ii^	111.78 (3)
Cl1—Zn1—Cl3^i^	109.47 (3)	N1—C12—C11	108.6 (3)
Cl2—Zn1—Cl3	116.80 (3)	N1—C12—C13	110.2 (3)
Cl2—Zn1—Cl3^i^	106.83 (3)	C11—C12—C13	114.0 (3)

Symmetry codes: (i) 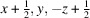; (ii) 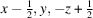.

**Table 5:** Database and calculated bond lengths (Å) and angles (°) for IPA^+^ and TMA^+^ cations

		C—C	C—N	C—C—C/C—N—C	C—C—N
IPA^+^					
	Mean (CSD)	1.50 (5)	1.49 (5)	114 (6)	110 (5)
	Calculated (*in vacuo*)	1.522	1.548	114.8	108.2
	Calculated (dielectric)	1.523	1.518	114.0	108.6
					
TMA^+^					
	Mean (CSD)		1.48 (5)	111 (5)	
	Calculated (*in vacuo*)		1.506	111.9	
	Calculated (dielectric)		1.498	111.7	

**Table 6:** Hydrogen-bond geometry (Å, °) for **I**[Chem scheme1]

*D*—H⋯*A*	*D*—H	H⋯*A*	*D*⋯*A*	*D*—H⋯*A*
N1—H1*A*⋯Cl1	0.89	2.43	3.321 (2)	178
N1—H1*B*⋯Cl3^i^	0.89	2.47	3.322 (2)	160
N1—H1*C*⋯Cl5^i^	0.89	2.51	3.400 (2)	173
N2—H2*A*⋯Cl2	0.89	2.58	3.274 (2)	136
N2—H2*A*⋯Cl4	0.89	2.83	3.537 (2)	137
N2—H2*B*⋯Cl3^ii^	0.89	2.46	3.339 (2)	169
N2—H2*C*⋯Cl5	0.89	2.44	3.282 (2)	157
N3—H3*A*⋯Cl5	0.89	2.35	3.236 (3)	173
N3—H3*B*⋯Cl5^iii^	0.89	2.31	3.197 (3)	173
N3—H3*C*⋯Cl4^iv^	0.89	2.75	3.374 (3)	128
C12—H12⋯Cl4^v^	0.98	2.81	3.676 (2)	148
C32—H32⋯Cl2	0.98	2.88	3.732 (4)	146

Symmetry codes: (i) 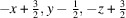; (ii) 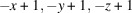; (iii) 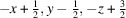; (iv) 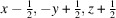; (v) 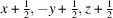.

**Table 7:** Hydrogen-bond geometry (Å, °) for **II**[Chem scheme1]

*D*—H⋯*A*	*D*—H	H⋯*A*	*D*⋯*A*	*D*—H⋯*A*
N1—H1*A*⋯Br3^i^	0.89	2.64	3.504 (4)	163
N1—H1*B*⋯Br5	0.89	2.72	3.611 (4)	175
N1—H1*C*⋯Br1^ii^	0.89	2.66	3.542 (4)	171
N2—H2*B*⋯Br3	0.89	2.65	3.499 (4)	160
N2—H2*A*⋯Br2^i^	0.89	2.70	3.445 (4)	143
N2—H2*A*⋯Br4^i^	0.89	2.99	3.607 (4)	128
N2—H2*C*⋯Br5	0.89	2.60	3.412 (4)	152
N3—H3*A*⋯Br5^iii^	0.89	2.51	3.380 (4)	165
N3—H3*B*⋯Br5	0.89	2.49	3.368 (4)	170
N3—H3*C*⋯Br4^iii^	0.89	3.01	3.538 (4)	119
C12—H12⋯Br4	0.98	2.85	3.751 (6)	154
C32—H32⋯Br2^i^	0.98	3.06	3.880 (5)	142

Symmetry codes: (i) 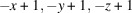; (ii) 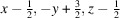; (iii) 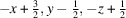.

**Table 8:** Hydrogen-bond geometry (Å, °) for **III**[Chem scheme1]

*D*—H⋯*A*	*D*—H	H⋯*A*	*D*⋯*A*	*D*—H⋯*A*
N1—H1*A*⋯Cl1	0.91 (6)	2.74 (6)	3.471 (4)	139 (4)
N1—H1*A*⋯Cl3	0.91 (6)	2.81 (6)	3.493 (4)	133 (4)
N1—H1*B*⋯Cl3^i^	0.79 (6)	2.51 (6)	3.287 (4)	166 (5)
N1—H1*C*⋯Cl1^ii^	0.88 (6)	2.46 (6)	3.321 (4)	167 (5)
N2—H2*A*⋯Cl1^iii^	1.00 (6)	2.68 (6)	3.400 (4)	129 (4)
N2—H2*A*⋯Cl2^iv^	1.00 (6)	2.53 (6)	3.346 (3)	139 (5)
N2—H2*B*⋯Cl2^v^	0.95 (5)	2.33 (5)	3.265 (4)	169 (4)
N2—H2*C*⋯Cl4	0.87 (5)	2.43 (5)	3.297 (4)	169 (4)

Symmetry codes: (i) 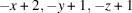; (ii) 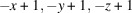; (iii) 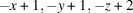; (iv) 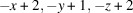; (v) 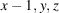.

**Table 9:** Hydrogen-bond geometry (Å, °) for **IV**[Chem scheme1]

*D*—H⋯*A*	*D*—H	H⋯*A*	*D*⋯*A*	*D*—H⋯*A*
N1—H1*A*⋯Cl2^iii^	0.84 (5)	2.47 (5)	3.292 (3)	165 (4)
N1—H1*B*⋯Cl1	0.88 (6)	2.81 (5)	3.317 (3)	118 (4)
N1—H1*B*⋯Cl1^ii^	0.88 (6)	2.74 (5)	3.356 (3)	128 (4)
N1—H1*C*⋯Cl1^i^	0.86 (5)	2.67 (5)	3.427 (3)	148 (4)
C12—H12⋯Cl2^i^	0.98	2.94	3.922 (4)	179

Symmetry codes: (i) 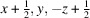; (ii) 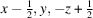; (iii) 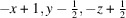.

**Table 10:** Experimental details

	**I**	**II**	**III**	**IV**
Crystal data				
Chemical formula	(C_3_H_10_N)_3_[CoCl_4_]Cl	(C_3_H_10_N)_3_[CoBr_4_]Br	(C_3_H_10_N)_2_[ZnCl_4_]	(C_3_H_10_N)[ZnCl_3_]
*M*_r_	416.54	638.84	327.41	231.84
Crystal system, space group	Monoclinic, *P*2_1_/*n*	Monoclinic, *P*2_1_/*n*	Monoclinic, *P*2_1_/*n*	Orthorhombic, *Pbca*
Temperature (K)	295	295	295	295
*a*, *b*, *c* (Å)	12.2051 (5), 9.5464 (4), 17.4681 (6)	12.7504 (5), 9.7339 (4), 17.8111 (7)	7.3076 (3), 20.4386 (8), 10.2616 (4)	6.3131 (3), 12.9582 (6), 21.8277 (10)
α, β, γ (°)	90, 91.360 (1), 90	90, 92.355 (2), 90	90, 107.827 (1), 90	90, 90, 90
*V* (Å^3^)	2034.72 (14)	2208.69 (15)	1459.05 (10)	1785.65 (14)
*Z*	4	4	4	8
Radiation type	Mo *K*α	Mo *K*α	Mo *K*α	Mo *K*α
μ (mm^−1^)	1.49	9.81	2.38	3.56
Crystal size (mm)	0.44 × 0.31 × 0.21	0.35 × 0.30 × 0.17	0.40 × 0.31 × 0.29	0.47 × 0.22 × 0.19

Data collection				
Diffractometer	Bruker D8 Quest Eco	Bruker D8 Quest Eco	Bruker D8 Quest Eco	Bruker D8 Quest Eco
Absorption correction	Multi-scan (*SADABS*; Krause *et al.*, 2015 ▸)	Multi-scan (*SADABS*; Krause *et al.*, 2015 ▸)	Multi-scan (*SADABS*; Krause *et al.*, 2015 ▸)	Multi-scan (*SADABS*; Krause *et al.*, 2015 ▸)
*T*_min_, *T*_max_	0.746, 0.886	0.528, 0.746	0.639, 0.746	0.444, 0.747
No. of measured, independent and observed [*I* > 2σ(*I*)] reflections	115887, 4663, 3721	88263, 5070, 3130	69211, 4105, 3568	137051, 3096, 2642
*R*_int_	0.052	0.097	0.075	0.041
(sin θ/λ)_max_ (Å^−1^)	0.650	0.650	0.696	0.746

Refinement				
*R*[*F*^2^ > 2σ(*F*^2^)], *wR*(*F*^2^), *S*	0.032, 0.076, 1.06	0.037, 0.065, 1.01	0.050, 0.105, 1.24	0.036, 0.076, 1.26
No. of reflections	4663	5070	4105	3096
No. of parameters	185	179	146	88
No. of restraints	6	3	0	0
H-atom treatment	H-atom parameters constrained	H-atom parameters constrained	H atoms treated by a mixture of independent and constrained refinement	H atoms treated by a mixture of independent and constrained refinement
Δρ_max_, Δρ_min_ (e Å^−3^)	0.51, −0.42	0.56, −0.50	0.64, −0.50	0.58, −0.74

Computer programs: *APEX3* v2017.3-0 (Bruker, 2017 ▸), *SAINT* v8.38A (Bruker, 2017 ▸), *SHELXT2018/2* (Sheldrick, 2015*a* ▸), *SHELXL2018/3* (Sheldrick, 2015*b* ▸), *ORTEP-3 for Windows* 2020.1 (Farrugia, 2012 ▸), *ORTEPIII* (Burnett & Johnson, 1996 ▸), *publCIF* (Westrip, 2010 ▸) and *PARST* (Nardelli, 1995 ▸), *publCIF* (Westrip, 2010 ▸).

## supplementary crystallographic information

### Tris(isopropylammonium) tetrachloridocobaltate(II) chloride (I) Crystal data

**Table:**

(C_3_H_10_N)_3_[CoCl_4_]Cl	*F*(000) = 868
*M**_r_* = 416.54	*D*_x_ = 1.36 Mg m^−^^3^
Monoclinic, *P*2_1_/*n*	Mo *K*α radiation, λ = 0.71073 Å
Hall symbol: -P 2yn	Cell parameters from 9894 reflections
*a* = 12.2051 (5) Å	θ = 5.4–56.2°
*b* = 9.5464 (4) Å	µ = 1.49 mm^−^^1^
*c* = 17.4681 (6) Å	*T* = 295 K
β = 91.360 (1)°	Irregular, blue
*V* = 2034.72 (14) Å^3^	0.44 × 0.31 × 0.21 mm
*Z* = 4	

### Tris(isopropylammonium) tetrachloridocobaltate(II) chloride (I) Data collection

**Table:**

Bruker D8 Quest Eco diffractometer	3721 reflections with *I* > 2σ(*I*)
φ and ω scans	*R*_int_ = 0.052
Absorption correction: multi-scan (SADABS; Krause *et al.*, 2015)	θ_max_ = 27.5°, θ_min_ = 3.3°
*T*_min_ = 0.746, *T*_max_ = 0.886	*h* = −15→15
115887 measured reflections	*k* = −12→12
4663 independent reflections	*l* = −22→22

### Tris(isopropylammonium) tetrachloridocobaltate(II) chloride (I) Refinement

**Table:**

Refinement on *F*^2^	Hydrogen site location: inferred from neighbouring sites
Least-squares matrix: full	H-atom parameters constrained
*R*[*F*^2^ > 2σ(*F*^2^)] = 0.032	*w* = 1/[σ^2^(*F*_o_^2^) + (0.028*P*)^2^ + 1.2921*P*] where *P* = (*F*_o_^2^ + 2*F*_c_^2^)/3
*wR*(*F*^2^) = 0.076	(Δ/σ)_max_ = 0.001
*S* = 1.06	Δρ_max_ = 0.51 e Å^−^^3^
4663 reflections	Δρ_min_ = −0.42 e Å^−^^3^
185 parameters	Extinction correction: SHELXL2018/3 (Sheldrick 2015b), Fc^*^=kFc[1+0.001xFc^2^λ^3^/sin(2θ)]^-1/4^
6 restraints	Extinction coefficient: 0.0154 (6)
Primary atom site location: dual	

### Tris(isopropylammonium) tetrachloridocobaltate(II) chloride (I) Special details

**Table:**

Geometry. All esds (except the esd in the dihedral angle between two l.s. planes) are estimated using the full covariance matrix. The cell esds are taken into account individually in the estimation of esds in distances, angles and torsion angles; correlations between esds in cell parameters are only used when they are defined by crystal symmetry. An approximate (isotropic) treatment of cell esds is used for estimating esds involving l.s. planes.
Refinement. DFIX restraints were applied to C-C and C-N bonds of minor disorder components in order to yield more chemically reasonable geometries. Four low angle reflections with Fc >> Fo were presumed to have been blocked by the beam catcher and were omitted from the refinement. APEX3 software suggested data collection to θmax = 31.66° however data analysis showed that <I/σ(I)> is less than 1.35 beyond θ = 27.5°. Thus data with θ > 27.5° were omitted from the final refinement.

### Tris(isopropylammonium) tetrachloridocobaltate(II) chloride (I) Fractional atomic coordinates and isotropic or equivalent isotropic displacement parameters (Å^2^)

**Table:**

	*x*	*y*	*z*	*U*_iso_*/*U*_eq_	Occ. (<1)
Co1	0.69716 (2)	0.32140 (3)	0.58630 (2)	0.03708 (10)	
Cl1	0.84051 (6)	0.17232 (7)	0.59286 (4)	0.0676 (2)	
Cl2	0.57605 (5)	0.28442 (8)	0.68007 (3)	0.05848 (17)	
Cl3	0.75183 (5)	0.54935 (6)	0.59658 (3)	0.05078 (15)	
Cl4	0.60820 (5)	0.30846 (7)	0.47051 (3)	0.05420 (16)	
Cl5	0.31607 (5)	0.51916 (6)	0.72833 (3)	0.04879 (15)	
N1	0.92624 (18)	0.1541 (2)	0.77442 (12)	0.0576 (5)	
H1A	0.901612	0.157398	0.726083	0.086*	
H1B	0.879586	0.105505	0.802408	0.086*	
H1C	0.991633	0.112882	0.776326	0.086*	
C11	1.0230 (3)	0.3748 (4)	0.7629 (3)	0.0976 (12)	
H11A	1.004113	0.376131	0.709216	0.146*	
H11B	1.091876	0.327744	0.770645	0.146*	
H11C	1.028876	0.469192	0.781518	0.146*	
C12	0.9361 (2)	0.2992 (3)	0.80528 (14)	0.0527 (6)	
H12	0.958964	0.293460	0.859340	0.063*	
C13	0.8279 (2)	0.3728 (3)	0.80011 (17)	0.0647 (7)	
H13A	0.833880	0.462314	0.824890	0.097*	
H13B	0.773476	0.317389	0.824861	0.097*	
H13C	0.806975	0.385861	0.747273	0.097*	
N2	0.36072 (16)	0.34578 (19)	0.56983 (12)	0.0487 (5)	0.847 (9)
H2A	0.433393	0.338721	0.573053	0.073*	0.847 (9)
H2B	0.340552	0.372232	0.522713	0.073*	0.847 (9)
H2C	0.338146	0.409058	0.603386	0.073*	0.847 (9)
C21	0.1882 (2)	0.2206 (4)	0.5815 (2)	0.0825 (10)	0.847 (9)
H21A	0.154769	0.132088	0.592536	0.124*	0.847 (9)
H21B	0.164498	0.289329	0.617668	0.124*	0.847 (9)
H21C	0.167094	0.249606	0.530650	0.124*	0.847 (9)
C22	0.3102 (2)	0.2063 (3)	0.5873 (2)	0.0493 (9)	0.847 (9)
H22	0.332145	0.176822	0.639137	0.059*	0.847 (9)
C23	0.3527 (5)	0.1028 (4)	0.5296 (4)	0.0962 (19)	0.847 (9)
H23A	0.431045	0.096386	0.534981	0.144*	0.847 (9)
H23B	0.320868	0.012464	0.538435	0.144*	0.847 (9)
H23C	0.333327	0.133799	0.478798	0.144*	0.847 (9)
N2A	0.36072 (16)	0.34578 (19)	0.56983 (12)	0.0487 (5)	0.153 (9)
H2D	0.319705	0.417888	0.554243	0.073*	0.153 (9)
H2E	0.371124	0.350132	0.620374	0.073*	0.153 (9)
H2F	0.425191	0.349063	0.547137	0.073*	0.153 (9)
C21A	0.1882 (2)	0.2206 (4)	0.5815 (2)	0.0825 (10)	0.153 (9)
H21D	0.152645	0.303460	0.562131	0.124*	0.153 (9)
H21E	0.146331	0.139642	0.566456	0.124*	0.153 (9)
H21F	0.193505	0.225238	0.636376	0.124*	0.153 (9)
C22A	0.3032 (8)	0.2103 (12)	0.5490 (12)	0.054 (6)*	0.153 (9)
H22A	0.295325	0.206653	0.493049	0.064*	0.153 (9)
C23A	0.369 (2)	0.086 (2)	0.5741 (16)	0.076 (7)*	0.153 (9)
H23D	0.330711	0.001540	0.560134	0.114*	0.153 (9)
H23E	0.438843	0.087348	0.549676	0.114*	0.153 (9)
H23F	0.380523	0.088501	0.628673	0.114*	0.153 (9)
N3	0.3157 (2)	0.2693 (3)	0.85354 (17)	0.0536 (9)	0.767 (5)
H3A	0.311029	0.341121	0.821145	0.080*	0.767 (5)
H3B	0.276986	0.197557	0.834767	0.080*	0.767 (5)
H3C	0.289183	0.294581	0.898517	0.080*	0.767 (5)
C31	0.4968 (2)	0.3532 (3)	0.89475 (18)	0.0705 (8)	0.767 (5)
H31A	0.572654	0.328655	0.901980	0.106*	0.767 (5)
H31B	0.490421	0.428970	0.858792	0.106*	0.767 (5)
H31C	0.467391	0.381537	0.942811	0.106*	0.767 (5)
C32	0.4333 (2)	0.2265 (3)	0.86397 (19)	0.0436 (8)	0.767 (5)
H32	0.462624	0.197537	0.814707	0.052*	0.767 (5)
C33	0.4364 (2)	0.1053 (3)	0.91902 (17)	0.0643 (7)	0.767 (5)
H33A	0.510851	0.075157	0.927024	0.096*	0.767 (5)
H33B	0.406784	0.133995	0.966964	0.096*	0.767 (5)
H33C	0.393628	0.029341	0.898200	0.096*	0.767 (5)
N3A	0.3791 (7)	0.2390 (9)	0.8083 (4)	0.046 (2)*	0.233 (5)
H3D	0.323163	0.180367	0.800785	0.069*	0.233 (5)
H3E	0.361927	0.322420	0.788680	0.069*	0.233 (5)
H3F	0.437994	0.205562	0.785396	0.069*	0.233 (5)
C31A	0.4968 (2)	0.3532 (3)	0.89475 (18)	0.0705 (8)	0.233 (5)
H31D	0.518483	0.369427	0.947191	0.106*	0.233 (5)
H31E	0.557297	0.314157	0.867793	0.106*	0.233 (5)
H31F	0.475247	0.440206	0.871335	0.106*	0.233 (5)
C32A	0.4027 (7)	0.2533 (8)	0.8915 (5)	0.040 (3)*	0.233 (5)
H32A	0.339421	0.289118	0.918912	0.049*	0.233 (5)
C33A	0.4364 (2)	0.1053 (3)	0.91902 (17)	0.0643 (7)	0.233 (5)
H33D	0.452948	0.107515	0.973013	0.096*	0.233 (5)
H33E	0.377238	0.041121	0.908982	0.096*	0.233 (5)
H33F	0.499980	0.075242	0.892161	0.096*	0.233 (5)

### Tris(isopropylammonium) tetrachloridocobaltate(II) chloride (I) Atomic displacement parameters (Å^2^)

**Table:**

	*U*^11^	*U*^22^	*U*^33^	*U*^12^	*U*^13^	*U*^23^
Co1	0.03859 (16)	0.03711 (16)	0.03567 (16)	0.00483 (12)	0.00363 (11)	0.00090 (11)
Cl1	0.0658 (4)	0.0634 (4)	0.0741 (4)	0.0324 (3)	0.0139 (3)	0.0086 (3)
Cl2	0.0478 (3)	0.0835 (5)	0.0446 (3)	0.0001 (3)	0.0107 (2)	0.0111 (3)
Cl3	0.0607 (3)	0.0403 (3)	0.0513 (3)	−0.0041 (2)	0.0021 (3)	−0.0033 (2)
Cl4	0.0646 (4)	0.0589 (4)	0.0387 (3)	−0.0102 (3)	−0.0067 (2)	−0.0019 (2)
Cl5	0.0511 (3)	0.0439 (3)	0.0512 (3)	0.0040 (2)	−0.0027 (2)	0.0026 (2)
N1	0.0614 (13)	0.0497 (12)	0.0622 (13)	0.0076 (10)	0.0102 (10)	0.0124 (10)
C11	0.071 (2)	0.073 (2)	0.150 (4)	−0.0119 (17)	0.032 (2)	0.012 (2)
C12	0.0533 (13)	0.0557 (15)	0.0490 (13)	0.0024 (11)	−0.0033 (10)	0.0065 (11)
C13	0.0638 (16)	0.0598 (16)	0.0705 (18)	0.0155 (13)	0.0017 (13)	0.0054 (14)
N2	0.0475 (10)	0.0354 (10)	0.0629 (12)	−0.0013 (8)	−0.0048 (9)	0.0005 (9)
C21	0.0636 (18)	0.075 (2)	0.109 (3)	−0.0168 (15)	0.0059 (17)	0.0235 (19)
C22	0.0631 (18)	0.0385 (16)	0.046 (2)	−0.0051 (12)	−0.0032 (13)	0.0077 (12)
C23	0.128 (4)	0.044 (2)	0.118 (4)	−0.014 (2)	0.041 (3)	−0.024 (2)
N2A	0.0475 (10)	0.0354 (10)	0.0629 (12)	−0.0013 (8)	−0.0048 (9)	0.0005 (9)
C21A	0.0636 (18)	0.075 (2)	0.109 (3)	−0.0168 (15)	0.0059 (17)	0.0235 (19)
N3	0.0455 (15)	0.0422 (14)	0.073 (2)	−0.0085 (11)	−0.0055 (13)	0.0159 (13)
C31	0.0641 (16)	0.0670 (18)	0.0797 (19)	−0.0243 (14)	−0.0168 (14)	0.0104 (15)
C32	0.0410 (15)	0.0508 (17)	0.0394 (16)	−0.0032 (13)	0.0091 (13)	0.0010 (13)
C33	0.0663 (16)	0.0546 (15)	0.0717 (18)	0.0039 (13)	−0.0019 (13)	0.0176 (13)
C31A	0.0641 (16)	0.0670 (18)	0.0797 (19)	−0.0243 (14)	−0.0168 (14)	0.0104 (15)
C33A	0.0663 (16)	0.0546 (15)	0.0717 (18)	0.0039 (13)	−0.0019 (13)	0.0176 (13)

### Tris(isopropylammonium) tetrachloridocobaltate(II) chloride (I) Geometric parameters (Å, º)

**Table:**

Co1—Cl1	2.2561 (6)	C21A—H21E	0.9600
Co1—Cl2	2.2600 (6)	C21A—H21F	0.9600
Co1—Cl3	2.2820 (6)	C22A—C23A	1.496 (10)
Co1—Cl4	2.2762 (6)	C22A—H22A	0.9800
N1—H1A	0.8900	C21A—C22A	1.529 (9)
N1—H1B	0.8900	C23A—H23D	0.9600
N1—H1C	0.8900	C23A—H23E	0.9600
N1—C12	1.490 (3)	C23A—H23F	0.9600
C11—H11A	0.9600	N3—H3A	0.8900
C11—H11B	0.9600	N3—H3B	0.8900
C11—H11C	0.9600	N3—H3C	0.8900
C11—C12	1.494 (4)	N3—C32	1.499 (4)
C12—C13	1.496 (3)	C31—H31A	0.9600
C12—H12	0.9800	C31—H31B	0.9600
C13—H13A	0.9600	C31—H31C	0.9600
C13—H13B	0.9600	C31—C32	1.527 (4)
C13—H13C	0.9600	C32—C33	1.505 (4)
N2—H2A	0.8900	C32—H32	0.9800
N2—H2B	0.8900	C33—H33A	0.9600
N2—H2C	0.8900	C33—H33B	0.9600
N2—C22	1.502 (3)	C33—H33C	0.9600
C21—H21A	0.9600	N3A—H3D	0.8900
C21—H21B	0.9600	N3A—H3E	0.8900
C21—H21C	0.9600	N3A—H3F	0.8900
C21—C22	1.496 (4)	N3A—C32A	1.482 (8)
C22—C23	1.512 (5)	C31A—H31D	0.9600
C22—H22	0.9800	C31A—H31E	0.9600
C23—H23A	0.9600	C31A—H31F	0.9600
C23—H23B	0.9600	C31A—C32A	1.493 (7)
C23—H23C	0.9600	C32A—C33A	1.545 (7)
N2A—H2D	0.8900	C32A—H32A	0.9800
N2A—H2E	0.8900	C33A—H33D	0.9600
N2A—H2F	0.8900	C33A—H33E	0.9600
N2A—C22A	1.512 (9)	C33A—H33F	0.9600
C21A—H21D	0.9600		
			
Cl1—Co1—Cl2	112.61 (3)	H21D—C21A—H21E	109.5
Cl1—Co1—Cl3	111.87 (3)	H21D—C21A—H21F	109.5
Cl1—Co1—Cl4	111.34 (3)	H21E—C21A—H21F	109.5
Cl2—Co1—Cl3	106.68 (3)	C22A—C21A—H21D	109.5
Cl2—Co1—Cl4	109.22 (3)	C22A—C21A—H21E	109.5
Cl3—Co1—Cl4	104.72 (2)	C22A—C21A—H21F	109.5
H1A—N1—H1B	109.5	C21A—C22A—H22A	107.7
C12—N1—H1A	109.5	C23A—C22A—H22A	107.7
H1A—N1—H1C	109.5	C21A—C22A—C23A	115.8 (16)
H1B—N1—H1C	109.5	C22A—C23A—H23D	109.5
C12—N1—H1B	109.5	C22A—C23A—H23E	109.5
C12—N1—H1C	109.5	C22A—C23A—H23F	109.5
N1—C12—C11	108.8 (2)	H23D—C23A—H23E	109.5
N1—C12—C13	110.6 (2)	H23D—C23A—H23F	109.5
N1—C12—H12	108.4	H23E—C23A—H23F	109.5
H11A—C11—H11B	109.5	H3A—N3—H3B	109.5
H11A—C11—H11C	109.5	H3A—N3—H3C	109.5
H11B—C11—H11C	109.5	H3B—N3—H3C	109.5
C12—C11—H11A	109.5	C32—N3—H3A	109.5
C12—C11—H11B	109.5	C32—N3—H3B	109.5
C12—C11—H11C	109.5	C32—N3—H3C	109.5
C11—C12—C13	112.3 (2)	N3—C32—C31	107.7 (3)
C11—C12—H12	108.4	N3—C32—C33	107.3 (2)
C13—C12—H12	108.4	N3—C32—H32	109.8
C12—C13—H13A	109.5	H31A—C31—H31B	109.5
C12—C13—H13B	109.5	H31A—C31—H31C	109.5
C12—C13—H13C	109.5	H31B—C31—H31C	109.5
H13A—C13—H13B	109.5	C32—C31—H31A	109.5
H13A—C13—H13C	109.5	C32—C31—H31B	109.5
H13B—C13—H13C	109.5	C32—C31—H31C	109.5
H2A—N2—H2C	109.5	C31—C32—H32	109.8
H2B—N2—H2C	109.5	C33—C32—H32	109.8
H2A—N2—H2B	109.5	C31—C32—C33	112.3 (3)
C22—N2—H2A	109.5	C32—C33—H33A	109.5
C22—N2—H2B	109.5	C32—C33—H33B	109.5
C22—N2—H2C	109.5	C32—C33—H33C	109.5
N2—C22—C21	108.5 (2)	H33A—C33—H33B	109.5
N2—C22—C23	107.2 (3)	H33A—C33—H33C	109.5
N2—C22—H22	109.7	H33B—C33—H33C	109.5
H21A—C21—H21B	109.5	H3D—N3A—H3E	109.5
H21A—C21—H21C	109.5	H3D—N3A—H3F	109.5
H21B—C21—H21C	109.5	H3E—N3A—H3F	109.5
C22—C21—H21A	109.5	C32A—N3A—H3D	109.5
C22—C21—H21B	109.5	C32A—N3A—H3E	109.5
C22—C21—H21C	109.5	C32A—N3A—H3F	109.5
C21—C22—H22	109.7	N3A—C32A—C31A	103.2 (6)
C23—C22—H22	109.7	N3A—C32A—C33A	105.3 (6)
C21—C22—C23	111.8 (4)	N3A—C32A—H32A	112.0
C22—C23—H23A	109.5	H31D—C31A—H31E	109.5
C22—C23—H23B	109.5	H31D—C31A—H31F	109.5
C22—C23—H23C	109.5	H31E—C31A—H31F	109.5
H23A—C23—H23B	109.5	C32A—C31A—H31D	109.5
H23A—C23—H23C	109.5	C32A—C31A—H31E	109.5
H23B—C23—H23C	109.5	C32A—C31A—H31F	109.5
H2D—N2A—H2E	109.5	C31A—C32A—H32A	112.0
H2D—N2A—H2F	109.5	C33A—C32A—H32A	112.0
H2E—N2A—H2F	109.5	C31A—C32A—C33A	111.9 (5)
C22A—N2A—H2D	109.5	C32A—C33A—H33D	109.5
C22A—N2A—H2E	109.5	C32A—C33A—H33E	109.5
C22A—N2A—H2F	109.5	C32A—C33A—H33F	109.5
N2A—C22A—C21A	106.3 (7)	H33D—C33A—H33E	109.5
N2A—C22A—C23A	111.4 (15)	H33D—C33A—H33F	109.5
N2A—C22A—H22A	107.7	H33E—C33A—H33F	109.5

### Tris(isopropylammonium) tetrachloridocobaltate(II) chloride (I) Hydrogen-bond geometry (Å, º)

**Table:**

*D*—H···*A*	*D*—H	H···*A*	*D*···*A*	*D*—H···*A*
N1—H1*A*···Cl1	0.89	2.43	3.321 (2)	178
N1—H1*B*···Cl3^i^	0.89	2.47	3.322 (2)	160
N1—H1*C*···Cl5^i^	0.89	2.51	3.400 (2)	173
N2—H2*A*···Cl2	0.89	2.58	3.274 (2)	136
N2—H2*A*···Cl4	0.89	2.83	3.537 (2)	137
N2—H2*B*···Cl3^ii^	0.89	2.46	3.339 (2)	169
N2—H2*C*···Cl5	0.89	2.44	3.282 (2)	157
N2*A*—H2*D*···Cl3^ii^	0.89	2.77	3.339 (2)	123
N2*A*—H2*D*···Cl4^ii^	0.89	2.79	3.3982 (19)	126
N2*A*—H2*E*···Cl5	0.89	2.58	3.282 (2)	136
N2*A*—H2*F*···Cl4	0.89	2.66	3.537 (2)	169
N3—H3*A*···Cl5	0.89	2.35	3.236 (3)	173
N3—H3*B*···Cl5^iii^	0.89	2.31	3.197 (3)	173
N3—H3*C*···Cl4^iv^	0.89	2.75	3.374 (3)	128
N3*A*—H3*D*···Cl5^iii^	0.89	2.34	3.227 (8)	175
N3*A*—H3*E*···Cl5	0.89	2.22	3.106 (8)	174
N3*A*—H3*F*···Cl2	0.89	2.63	3.352 (8)	138
C12—H12···Cl4^v^	0.98	2.81	3.676 (2)	148
C32—H32···Cl2	0.98	2.88	3.732 (4)	146

Symmetry codes: (i) −*x*+3/2, *y*−1/2, −*z*+3/2; (ii) −*x*+1, −*y*+1, −*z*+1; (iii) −*x*+1/2, *y*−1/2, −*z*+3/2; (iv) *x*−1/2, −*y*+1/2, *z*+1/2; (v) *x*+1/2, −*y*+1/2, *z*+1/2.

### Tris(isopropylammonium) tetrabromidocobaltate(II) bromide (II) Crystal data

**Table:**

(C_3_H_10_N)_3_[CoBr_4_]Br	*F*(000) = 1228
*M**_r_* = 638.84	*D*_x_ = 1.921 Mg m^−^^3^
Monoclinic, *P*2_1_/*n*	Mo *K*α radiation, λ = 0.71073 Å
Hall symbol: -P 2yn	Cell parameters from 9847 reflections
*a* = 12.7504 (5) Å	θ = 5.3–46.9°
*b* = 9.7339 (4) Å	µ = 9.81 mm^−^^1^
*c* = 17.8111 (7) Å	*T* = 295 K
β = 92.355 (2)°	Irregular, blue
*V* = 2208.69 (15) Å^3^	0.35 × 0.30 × 0.17 mm
*Z* = 4	

### Tris(isopropylammonium) tetrabromidocobaltate(II) bromide (II) Data collection

**Table:**

Bruker D8 Quest Eco diffractometer	3130 reflections with *I* > 2σ(*I*)
φ and ω scans	*R*_int_ = 0.097
Absorption correction: multi-scan (SADABS; Krause *et al.*, 2015)	θ_max_ = 27.5°, θ_min_ = 3.1°
*T*_min_ = 0.528, *T*_max_ = 0.746	*h* = −16→16
88263 measured reflections	*k* = −12→12
5070 independent reflections	*l* = −23→23

### Tris(isopropylammonium) tetrabromidocobaltate(II) bromide (II) Refinement

**Table:**

Refinement on *F*^2^	Hydrogen site location: inferred from neighbouring sites
Least-squares matrix: full	H-atom parameters constrained
*R*[*F*^2^ > 2σ(*F*^2^)] = 0.037	*w* = 1/[σ^2^(*F*_o_^2^) + (0.0135*P*)^2^ + 4.4182*P*] where *P* = (*F*_o_^2^ + 2*F*_c_^2^)/3
*wR*(*F*^2^) = 0.065	(Δ/σ)_max_ = 0.001
*S* = 1.01	Δρ_max_ = 0.56 e Å^−^^3^
5070 reflections	Δρ_min_ = −0.50 e Å^−^^3^
179 parameters	Extinction correction: SHELXL-2018/3 (Sheldrick 2015b), Fc^*^=kFc[1+0.001xFc^2^λ^3^/sin(2θ)]^-1/4^
3 restraints	Extinction coefficient: 0.00346 (11)
Primary atom site location: dual	

### Tris(isopropylammonium) tetrabromidocobaltate(II) bromide (II) Special details

**Table:**

Geometry. All esds (except the esd in the dihedral angle between two l.s. planes) are estimated using the full covariance matrix. The cell esds are taken into account individually in the estimation of esds in distances, angles and torsion angles; correlations between esds in cell parameters are only used when they are defined by crystal symmetry. An approximate (isotropic) treatment of cell esds is used for estimating esds involving l.s. planes.
Refinement. DFIX restraints were applied to C-C and C-N bonds of the minor disorder component in order to yield a more chemically reasonable geometry. Four low angle reflections with Fc >> Fo were presumed to have been blocked by the beam catcher and were omitted from the refinement. APEX3 software suggested data collection to θmax = 28.32° however data analysis showed that <I/σ(I)> is less than 1.03 beyond θ = 27.5°. Thus data with θ > 27.5° were omitted from the final refinement.

### Tris(isopropylammonium) tetrabromidocobaltate(II) bromide (II) Fractional atomic coordinates and isotropic or equivalent isotropic displacement parameters (Å^2^)

**Table:**

	*x*	*y*	*z*	*U*_iso_*/*U*_eq_	Occ. (<1)
Co1	0.69245 (4)	0.67873 (6)	0.58622 (3)	0.03968 (16)	
Br1	0.83023 (4)	0.84556 (6)	0.58385 (3)	0.07107 (18)	
Br2	0.57849 (4)	0.71422 (6)	0.68763 (3)	0.05936 (16)	
Br3	0.75948 (4)	0.44794 (5)	0.59845 (3)	0.05384 (15)	
Br4	0.59477 (4)	0.68488 (5)	0.46746 (3)	0.05525 (15)	
Br5	0.68875 (4)	0.51863 (5)	0.26865 (3)	0.05417 (15)	
N1	0.4261 (3)	0.6561 (4)	0.2734 (2)	0.0700 (12)	
H1A	0.387031	0.612385	0.306060	0.105*	
H1B	0.488965	0.616607	0.272491	0.105*	
H1C	0.394878	0.651897	0.227802	0.105*	
C11	0.5163 (5)	0.8676 (7)	0.2475 (5)	0.119 (3)	
H11A	0.582585	0.821337	0.253886	0.178*	
H11B	0.524627	0.962415	0.261344	0.178*	
H11C	0.491953	0.861520	0.195882	0.178*	
C12	0.4383 (4)	0.8016 (6)	0.2962 (3)	0.0760 (16)	
H12	0.469908	0.801058	0.347336	0.091*	
C13	0.3397 (4)	0.8734 (6)	0.3000 (4)	0.0862 (19)	
H13A	0.292873	0.821329	0.329812	0.129*	
H13B	0.309203	0.884725	0.250229	0.129*	
H13C	0.351462	0.961995	0.322536	0.129*	
N2	0.6409 (3)	0.3389 (4)	0.4281 (2)	0.0514 (10)	0.735 (18)
H2A	0.571676	0.334733	0.419948	0.077*	0.735 (18)
H2B	0.655550	0.359392	0.476060	0.077*	0.735 (18)
H2C	0.667335	0.403361	0.398883	0.077*	0.735 (18)
C21	0.8050 (4)	0.2110 (6)	0.4240 (4)	0.0846 (19)	0.735 (18)
H21A	0.836205	0.124070	0.412618	0.127*	0.735 (18)
H21B	0.833120	0.280857	0.392525	0.127*	0.735 (18)
H21C	0.820408	0.233430	0.475772	0.127*	0.735 (18)
C22	0.6886 (5)	0.2026 (7)	0.4101 (7)	0.057 (3)	0.735 (18)
H22	0.671965	0.178198	0.357558	0.068*	0.735 (18)
C23	0.6411 (9)	0.0987 (10)	0.4619 (9)	0.108 (5)	0.735 (18)
H23A	0.566526	0.094872	0.452164	0.162*	0.735 (18)
H23B	0.671012	0.009867	0.453384	0.162*	0.735 (18)
H23C	0.655539	0.125456	0.513212	0.162*	0.735 (18)
N2A	0.6409 (3)	0.3389 (4)	0.4281 (2)	0.0514 (10)	0.265 (18)
H2D	0.679737	0.408126	0.446358	0.077*	0.265 (18)
H2E	0.634447	0.345965	0.378281	0.077*	0.265 (18)
H2F	0.577688	0.341785	0.447357	0.077*	0.265 (18)
C21A	0.8050 (4)	0.2110 (6)	0.4240 (4)	0.0846 (19)	0.265 (18)
H21D	0.839173	0.290545	0.445457	0.127*	0.265 (18)
H21E	0.841824	0.129778	0.440654	0.127*	0.265 (18)
H21F	0.805155	0.216464	0.370188	0.127*	0.265 (18)
C22A	0.6932 (9)	0.2050 (13)	0.4487 (14)	0.063 (9)*	0.265 (18)
H22A	0.695959	0.198427	0.503652	0.076*	0.265 (18)
C23A	0.631 (2)	0.085 (3)	0.4190 (15)	0.067 (8)*	0.265 (18)
H23D	0.665885	0.001244	0.433085	0.101*	0.265 (18)
H23E	0.562295	0.086328	0.439779	0.101*	0.265 (18)
H23F	0.623519	0.090836	0.365270	0.101*	0.265 (18)
N3	0.6836 (3)	0.2724 (4)	0.1357 (2)	0.0678 (12)	
H3A	0.724812	0.205045	0.153399	0.102*	
H3B	0.689121	0.344526	0.166351	0.102*	
H3C	0.703195	0.296527	0.090156	0.102*	
C31	0.5040 (4)	0.3426 (6)	0.1056 (3)	0.0708 (16)	
H31A	0.517748	0.365418	0.054465	0.106*	
H31B	0.518158	0.420890	0.137108	0.106*	
H31C	0.431723	0.316343	0.108870	0.106*	
C32	0.5736 (4)	0.2247 (5)	0.1311 (3)	0.0527 (12)	
H32	0.553744	0.196600	0.181377	0.063*	
C33	0.5661 (4)	0.1015 (5)	0.0802 (3)	0.0687 (15)	
H33A	0.584103	0.127780	0.030348	0.103*	
H33B	0.495727	0.066353	0.078975	0.103*	
H33C	0.613765	0.031802	0.098641	0.103*	

### Tris(isopropylammonium) tetrabromidocobaltate(II) bromide (II) Atomic displacement parameters (Å^2^)

**Table:**

	*U*^11^	*U*^22^	*U*^33^	*U*^12^	*U*^13^	*U*^23^
Co1	0.0428 (3)	0.0400 (3)	0.0366 (3)	−0.0033 (3)	0.0061 (3)	0.0000 (3)
Br1	0.0720 (4)	0.0633 (4)	0.0790 (4)	−0.0308 (3)	0.0165 (3)	−0.0057 (3)
Br2	0.0573 (3)	0.0759 (4)	0.0460 (3)	0.0013 (3)	0.0161 (2)	−0.0079 (3)
Br3	0.0621 (3)	0.0450 (3)	0.0545 (3)	0.0077 (2)	0.0034 (2)	0.0020 (2)
Br4	0.0658 (3)	0.0610 (3)	0.0386 (3)	0.0072 (3)	−0.0031 (2)	0.0026 (2)
Br5	0.0606 (3)	0.0532 (3)	0.0487 (3)	−0.0062 (2)	0.0026 (2)	−0.0029 (2)
N1	0.084 (3)	0.053 (3)	0.075 (3)	0.006 (2)	0.020 (2)	0.013 (2)
C11	0.079 (5)	0.098 (5)	0.183 (8)	−0.010 (4)	0.047 (5)	0.031 (5)
C12	0.075 (4)	0.065 (4)	0.088 (4)	−0.003 (3)	0.000 (3)	0.008 (3)
C13	0.084 (4)	0.064 (4)	0.112 (5)	0.018 (3)	0.026 (4)	0.013 (4)
N2	0.053 (2)	0.039 (2)	0.062 (3)	0.0039 (18)	0.002 (2)	0.0024 (19)
C21	0.064 (4)	0.087 (5)	0.102 (5)	0.022 (3)	0.000 (3)	−0.025 (4)
C22	0.066 (5)	0.044 (5)	0.059 (6)	0.010 (3)	−0.007 (4)	−0.014 (4)
C23	0.132 (9)	0.052 (6)	0.142 (12)	0.018 (6)	0.033 (9)	0.029 (7)
N2A	0.053 (2)	0.039 (2)	0.062 (3)	0.0039 (18)	0.002 (2)	0.0024 (19)
C21A	0.064 (4)	0.087 (5)	0.102 (5)	0.022 (3)	0.000 (3)	−0.025 (4)
N3	0.062 (3)	0.053 (3)	0.087 (3)	0.008 (2)	−0.005 (2)	−0.017 (2)
C31	0.057 (3)	0.078 (4)	0.077 (4)	0.020 (3)	−0.007 (3)	−0.010 (3)
C32	0.045 (3)	0.056 (3)	0.057 (3)	0.006 (2)	0.010 (2)	−0.001 (2)
C33	0.074 (4)	0.056 (3)	0.076 (4)	−0.011 (3)	0.008 (3)	−0.017 (3)

### Tris(isopropylammonium) tetrabromidocobaltate(II) bromide (II) Geometric parameters (Å, º)

**Table:**

Co1—Br1	2.3939 (7)	C23—H23B	0.9600
Co1—Br2	2.3894 (7)	C23—H23C	0.9600
Co1—Br3	2.4102 (7)	N2A—H2D	0.8900
Co1—Br4	2.4110 (7)	N2A—H2E	0.8900
N1—H1A	0.8900	N2A—H2F	0.8900
N1—H1B	0.8900	N2A—C22A	1.502 (9)
N1—H1C	0.8900	C21A—H21D	0.9600
N1—C12	1.480 (7)	C21A—H21E	0.9600
C11—H11A	0.9600	C21A—H21F	0.9600
C11—H11B	0.9600	C21A—C22A	1.510 (9)
C11—H11C	0.9600	C22A—H22A	0.9800
C11—C12	1.492 (8)	C22A—C23A	1.498 (10)
C12—H12	0.9800	C23A—H23D	0.9600
C12—C13	1.443 (7)	C23A—H23E	0.9600
C13—H13A	0.9600	C23A—H23F	0.9600
C13—H13B	0.9600	N3—H3A	0.8900
C13—H13C	0.9600	N3—H3B	0.8900
N2—H2A	0.8900	N3—H3C	0.8900
N2—H2B	0.8900	N3—C32	1.477 (6)
N2—H2C	0.8900	C31—H31A	0.9600
N2—C22	1.499 (7)	C31—H31B	0.9600
C21—H21A	0.9600	C31—H31C	0.9600
C21—H21B	0.9600	C31—C32	1.509 (6)
C21—H21C	0.9600	C32—H32	0.9800
C21—C22	1.498 (8)	C32—C33	1.504 (6)
C22—H22	0.9800	C33—H33A	0.9600
C22—C23	1.513 (13)	C33—H33B	0.9600
C23—H23A	0.9600	C33—H33C	0.9600
			
Br1—Co1—Br2	112.63 (3)	H23A—C23—H23B	109.5
Br1—Co1—Br3	112.09 (3)	H23A—C23—H23C	109.5
Br1—Co1—Br4	108.70 (3)	H23B—C23—H23C	109.5
Br2—Co1—Br3	106.91 (3)	H2D—N2A—H2E	109.5
Br2—Co1—Br4	110.57 (3)	H2D—N2A—H2F	109.5
Br3—Co1—Br4	105.72 (3)	H2E—N2A—H2F	109.5
H1A—N1—H1B	109.5	C22A—N2A—H2D	109.5
H1A—N1—H1C	109.5	C22A—N2A—H2E	109.5
H1B—N1—H1C	109.5	C22A—N2A—H2F	109.5
C12—N1—H1A	109.5	N2A—C22A—C21A	108.0 (8)
C12—N1—H1B	109.5	N2A—C22A—C23A	111.4 (18)
C12—N1—H1C	109.5	N2A—C22A—H22A	107.3
N1—C12—C11	108.5 (5)	H21D—C21A—H21E	109.5
N1—C12—C13	113.2 (5)	H21D—C21A—H21F	109.5
N1—C12—H12	106.5	H21E—C21A—H21F	109.5
H11A—C11—H11B	109.5	C22A—C21A—H21D	109.5
H11A—C11—H11C	109.5	C22A—C21A—H21E	109.5
H11B—C11—H11C	109.5	C22A—C21A—H21F	109.5
C12—C11—H11A	109.5	C21A—C22A—H22A	107.3
C12—C11—H11B	109.5	C23A—C22A—H22A	107.3
C12—C11—H11C	109.5	C21A—C22A—C23A	115.0 (18)
C11—C12—H12	106.5	C22A—C23A—H23D	109.5
C13—C12—H12	106.5	C22A—C23A—H23E	109.5
C13—C12—C11	114.9 (5)	C22A—C23A—H23F	109.5
C12—C13—H13A	109.5	H23D—C23A—H23E	109.5
C12—C13—H13B	109.5	H23D—C23A—H23F	109.5
C12—C13—H13C	109.5	H23E—C23A—H23F	109.5
H13A—C13—H13B	109.5	H3A—N3—H3B	109.5
H13A—C13—H13C	109.5	H3A—N3—H3C	109.5
H13B—C13—H13C	109.5	H3B—N3—H3C	109.5
H2A—N2—H2B	109.5	C32—N3—H3A	109.5
H2A—N2—H2C	109.5	C32—N3—H3B	109.5
H2B—N2—H2C	109.5	C32—N3—H3C	109.5
C22—N2—H2A	109.5	N3—C32—C31	108.8 (4)
C22—N2—H2B	109.5	N3—C32—C33	108.7 (4)
C22—N2—H2C	109.5	N3—C32—H32	108.5
N2—C22—C21	108.9 (5)	H31A—C31—H31B	109.5
N2—C22—C23	106.6 (8)	H31A—C31—H31C	109.5
N2—C22—H22	110.2	H31B—C31—H31C	109.5
H21A—C21—H21B	109.5	C32—C31—H31A	109.5
H21A—C21—H21C	109.5	C32—C31—H31B	109.5
H21B—C21—H21C	109.5	C32—C31—H31C	109.5
C22—C21—H21A	109.5	C31—C32—H32	108.5
C22—C21—H21B	109.5	C33—C32—H32	108.5
C22—C21—H21C	109.5	C31—C32—C33	113.8 (4)
C21—C22—H22	110.2	C32—C33—H33A	109.5
C23—C22—H22	110.2	C32—C33—H33B	109.5
C21—C22—C23	110.8 (9)	C32—C33—H33C	109.5
C22—C23—H23A	109.5	H33A—C33—H33B	109.5
C22—C23—H23B	109.5	H33A—C33—H33C	109.5
C22—C23—H23C	109.5	H33B—C33—H33C	109.5

### Tris(isopropylammonium) tetrabromidocobaltate(II) bromide (II) Hydrogen-bond geometry (Å, º)

**Table:**

*D*—H···*A*	*D*—H	H···*A*	*D*···*A*	*D*—H···*A*
N1—H1*A*···Br3^i^	0.89	2.64	3.504 (4)	163
N1—H1*B*···Br5	0.89	2.72	3.611 (4)	175
N1—H1*C*···Br1^ii^	0.89	2.66	3.542 (4)	171
N2—H2*B*···Br3	0.89	2.65	3.499 (4)	160
N2—H2*A*···Br2^i^	0.89	2.70	3.445 (4)	143
N2—H2*A*···Br4^i^	0.89	2.99	3.607 (4)	128
N2—H2*C*···Br5	0.89	2.60	3.412 (4)	152
N2*A*—H2*E*···Br5	0.89	2.69	3.412 (4)	139
N2*A*—H2*D*···Br3	0.89	2.88	3.499 (4)	128
N2*A*—H2*D*···Br4	0.89	2.93	3.495 (3)	123
N2*A*—H2*E*···Br2^i^	0.89	2.97	3.445 (4)	115
N2*A*—H2*F*···Br4^i^	0.89	2.73	3.607 (4)	167
N3—H3*A*···Br5^iii^	0.89	2.51	3.380 (4)	165
N3—H3*B*···Br5	0.89	2.49	3.368 (4)	170
N3—H3*C*···Br4^iii^	0.89	3.01	3.538 (4)	119
C12—H12···Br4	0.98	2.85	3.751 (6)	154
C32—H32···Br2^i^	0.98	3.06	3.880 (5)	142

Symmetry codes: (i) −*x*+1, −*y*+1, −*z*+1; (ii) *x*−1/2, −*y*+3/2, *z*−1/2; (iii) −*x*+3/2, *y*−1/2, −*z*+1/2.

### Bis(isopropylammonium) tetrachloridozincate(II) (III) Crystal data

**Table:**

(C_3_H_10_N)_2_[ZnCl_4_]	*F*(000) = 672
*M**_r_* = 327.41	*D*_x_ = 1.49 Mg m^−^^3^
Monoclinic, *P*2_1_/*n*	Mo *K*α radiation, λ = 0.71073 Å
Hall symbol: -P 2yn	Cell parameters from 9690 reflections
*a* = 7.3076 (3) Å	θ = 5.8–59.0°
*b* = 20.4386 (8) Å	µ = 2.38 mm^−^^1^
*c* = 10.2616 (4) Å	*T* = 295 K
β = 107.827 (1)°	Block, colourless
*V* = 1459.05 (10) Å^3^	0.40 × 0.31 × 0.29 mm
*Z* = 4	

### Bis(isopropylammonium) tetrachloridozincate(II) (III) Data collection

**Table:**

Bruker D8 Quest Eco diffractometer	3568 reflections with *I* > 2σ(*I*)
φ and ω scans	*R*_int_ = 0.075
Absorption correction: multi-scan (SADABS; Krause *et al.*, 2015)	θ_max_ = 29.6°, θ_min_ = 3.0°
*T*_min_ = 0.639, *T*_max_ = 0.746	*h* = −10→10
69211 measured reflections	*k* = −28→28
4105 independent reflections	*l* = −14→14

### Bis(isopropylammonium) tetrachloridozincate(II) (III) Refinement

**Table:**

Refinement on *F*^2^	Primary atom site location: dual
Least-squares matrix: full	Hydrogen site location: mixed
*R*[*F*^2^ > 2σ(*F*^2^)] = 0.050	H atoms treated by a mixture of independent and constrained refinement
*wR*(*F*^2^) = 0.105	*w* = 1/[σ^2^(*F*_o_^2^) + (0.0206*P*)^2^ + 2.6728*P*] where *P* = (*F*_o_^2^ + 2*F*_c_^2^)/3
*S* = 1.24	(Δ/σ)_max_ = 0.001
4105 reflections	Δρ_max_ = 0.64 e Å^−^^3^
146 parameters	Δρ_min_ = −0.50 e Å^−^^3^
0 restraints	

### Bis(isopropylammonium) tetrachloridozincate(II) (III) Special details

**Table:**

Geometry. All esds (except the esd in the dihedral angle between two l.s. planes) are estimated using the full covariance matrix. The cell esds are taken into account individually in the estimation of esds in distances, angles and torsion angles; correlations between esds in cell parameters are only used when they are defined by crystal symmetry. An approximate (isotropic) treatment of cell esds is used for estimating esds involving l.s. planes.
Refinement. Two low angle reflections with Fc >> Fo were presumed to have been blocked by the beam catcher and were omitted from the refinement.

### Bis(isopropylammonium) tetrachloridozincate(II) (III) Fractional atomic coordinates and isotropic or equivalent isotropic displacement parameters (Å^2^)

**Table:**

	*x*	*y*	*z*	*U*_iso_*/*U*_eq_	
Zn1	0.87245 (5)	0.57264 (2)	0.77526 (4)	0.03468 (11)	
Cl1	0.61651 (11)	0.50404 (4)	0.75532 (9)	0.03846 (18)	
Cl2	1.14947 (12)	0.51445 (5)	0.82326 (11)	0.0531 (2)	
Cl3	0.83177 (14)	0.60429 (6)	0.55485 (11)	0.0588 (3)	
Cl4	0.86591 (15)	0.65416 (5)	0.91889 (13)	0.0632 (3)	
N1	0.7569 (6)	0.43706 (17)	0.4900 (4)	0.0471 (7)	
H1A	0.772 (8)	0.469 (3)	0.555 (6)	0.086 (18)*	
H1B	0.852 (8)	0.432 (3)	0.469 (5)	0.072 (17)*	
H1C	0.672 (8)	0.456 (3)	0.421 (6)	0.076 (16)*	
C11	0.8492 (6)	0.3487 (2)	0.6582 (4)	0.0582 (10)	
H11A	0.964336	0.340844	0.634118	0.087*	
H11B	0.807838	0.308596	0.689201	0.087*	
H11C	0.874523	0.380662	0.730045	0.087*	
C12	0.6944 (5)	0.37347 (18)	0.5352 (4)	0.0419 (7)	
H12	0.578190	0.381280	0.561575	0.050*	
C13	0.6450 (7)	0.3269 (2)	0.4154 (5)	0.0621 (11)	
H13A	0.756303	0.320013	0.386063	0.093*	
H13B	0.543383	0.345116	0.341412	0.093*	
H13C	0.603904	0.285848	0.442470	0.093*	
N2	0.5139 (5)	0.59521 (16)	1.0224 (4)	0.0466 (7)	
H2A	0.566 (9)	0.562 (3)	1.095 (7)	0.11 (2)*	
H2B	0.420 (7)	0.570 (2)	0.958 (5)	0.073 (15)*	
H2C	0.605 (7)	0.606 (2)	0.987 (5)	0.059 (14)*	
C21	0.3642 (6)	0.7020 (2)	0.9601 (5)	0.0580 (11)	
H21A	0.472466	0.716289	0.933174	0.087*	
H21B	0.308334	0.738912	0.991874	0.087*	
H21C	0.270247	0.682572	0.883172	0.087*	
C22	0.4290 (5)	0.65261 (17)	1.0730 (4)	0.0397 (7)	
H22	0.316762	0.637783	1.098095	0.048*	
C23	0.5761 (7)	0.6788 (3)	1.1992 (4)	0.0663 (12)	
H23A	0.690318	0.690852	1.177432	0.099*	
H23B	0.606694	0.645778	1.269043	0.099*	
H23C	0.524975	0.716580	1.231653	0.099*	

### Bis(isopropylammonium) tetrachloridozincate(II) (III) Atomic displacement parameters (Å^2^)

**Table:**

	*U*^11^	*U*^22^	*U*^33^	*U*^12^	*U*^13^	*U*^23^
Zn1	0.02608 (17)	0.0375 (2)	0.0432 (2)	0.00084 (14)	0.01469 (14)	−0.00365 (16)
Cl1	0.0316 (4)	0.0402 (4)	0.0467 (4)	−0.0045 (3)	0.0168 (3)	0.0024 (3)
Cl2	0.0306 (4)	0.0554 (5)	0.0701 (6)	0.0137 (4)	0.0106 (4)	0.0022 (4)
Cl3	0.0413 (5)	0.0864 (7)	0.0550 (6)	0.0124 (5)	0.0241 (4)	0.0282 (5)
Cl4	0.0531 (6)	0.0561 (6)	0.0907 (8)	−0.0125 (4)	0.0371 (6)	−0.0363 (6)
N1	0.0476 (19)	0.0413 (17)	0.054 (2)	0.0073 (14)	0.0184 (16)	−0.0016 (15)
C11	0.055 (2)	0.071 (3)	0.051 (2)	0.013 (2)	0.0190 (19)	0.012 (2)
C12	0.0334 (16)	0.0469 (19)	0.050 (2)	0.0041 (14)	0.0190 (15)	0.0004 (15)
C13	0.065 (3)	0.049 (2)	0.067 (3)	0.005 (2)	0.010 (2)	−0.010 (2)
N2	0.0476 (18)	0.0360 (15)	0.054 (2)	0.0028 (14)	0.0120 (16)	0.0024 (14)
C21	0.053 (2)	0.045 (2)	0.072 (3)	0.0089 (17)	0.015 (2)	0.0154 (19)
C22	0.0382 (17)	0.0396 (17)	0.0452 (19)	−0.0032 (14)	0.0186 (14)	0.0025 (14)
C23	0.076 (3)	0.075 (3)	0.047 (2)	−0.018 (2)	0.017 (2)	−0.011 (2)

### Bis(isopropylammonium) tetrachloridozincate(II) (III) Geometric parameters (Å, º)

**Table:**

Zn1—Cl1	2.2960 (8)	C13—H13B	0.9600
Zn1—Cl2	2.2681 (9)	C13—H13C	0.9600
Zn1—Cl3	2.2828 (10)	N2—H2A	1.00 (6)
Zn1—Cl4	2.2347 (10)	N2—H2B	0.95 (5)
N1—H1A	0.91 (6)	N2—H2C	0.87 (5)
N1—H1B	0.79 (6)	N2—C22	1.493 (5)
N1—H1C	0.88 (6)	C21—H21A	0.9600
N1—C12	1.498 (5)	C21—H21B	0.9600
C11—H11A	0.9600	C21—H21C	0.9600
C11—H11B	0.9600	C21—C22	1.500 (5)
C11—H11C	0.9600	C22—H22	0.9800
C11—C12	1.502 (5)	C22—C23	1.505 (5)
C12—H12	0.9800	C23—H23A	0.9600
C12—C13	1.509 (5)	C23—H23B	0.9600
C13—H13A	0.9600	C23—H23C	0.9600
			
Cl1—Zn1—Cl2	110.35 (4)	H13A—C13—H13B	109.5
Cl1—Zn1—Cl3	103.26 (4)	H13A—C13—H13C	109.5
Cl1—Zn1—Cl4	109.44 (4)	H13B—C13—H13C	109.5
Cl2—Zn1—Cl3	101.57 (4)	H2A—N2—H2B	101 (4)
Cl2—Zn1—Cl4	116.46 (4)	H2A—N2—H2C	108 (4)
Cl3—Zn1—Cl4	114.81 (5)	H2B—N2—H2C	109 (4)
H1A—N1—H1B	112 (5)	C22—N2—H2A	112 (4)
H1A—N1—H1C	100 (4)	C22—N2—H2B	112 (3)
H1B—N1—H1C	107 (5)	C22—N2—H2C	113 (3)
C12—N1—H1A	112 (3)	N2—C22—C21	109.1 (3)
C12—N1—H1B	110 (4)	N2—C22—H22	108.6
C12—N1—H1C	116 (3)	N2—C22—C23	108.4 (3)
N1—C12—C11	109.3 (3)	H21A—C21—H21B	109.5
N1—C12—H12	108.4	H21A—C21—H21C	109.5
N1—C12—C13	108.3 (3)	H21B—C21—H21C	109.5
H11A—C11—H11B	109.5	C22—C21—H21A	109.5
H11A—C11—H11C	109.5	C22—C21—H21B	109.5
H11B—C11—H11C	109.5	C22—C21—H21C	109.5
C12—C11—H11A	109.5	C21—C22—H22	108.6
C12—C11—H11B	109.5	C23—C22—H22	108.6
C12—C11—H11C	109.5	C21—C22—C23	113.4 (3)
C11—C12—C13	113.7 (3)	C22—C23—H23A	109.5
C11—C12—H12	108.4	C22—C23—H23B	109.5
C13—C12—H12	108.4	C22—C23—H23C	109.5
C12—C13—H13A	109.5	H23A—C23—H23B	109.5
C12—C13—H13B	109.5	H23A—C23—H23C	109.5
C12—C13—H13C	109.5	H23B—C23—H23C	109.5

### Bis(isopropylammonium) tetrachloridozincate(II) (III) Hydrogen-bond geometry (Å, º)

**Table:**

*D*—H···*A*	*D*—H	H···*A*	*D*···*A*	*D*—H···*A*
N1—H1*A*···Cl1	0.91 (6)	2.74 (6)	3.471 (4)	139 (4)
N1—H1*A*···Cl3	0.91 (6)	2.81 (6)	3.493 (4)	133 (4)
N1—H1*B*···Cl3^i^	0.79 (6)	2.51 (6)	3.287 (4)	166 (5)
N1—H1*C*···Cl1^ii^	0.88 (6)	2.46 (6)	3.321 (4)	167 (5)
N2—H2*A*···Cl1^iii^	1.00 (6)	2.68 (6)	3.400 (4)	129 (4)
N2—H2*A*···Cl2^iv^	1.00 (6)	2.53 (6)	3.346 (3)	139 (5)
N2—H2*B*···Cl2^v^	0.95 (5)	2.33 (5)	3.265 (4)	169 (4)
N2—H2*C*···Cl4	0.87 (5)	2.43 (5)	3.297 (4)	169 (4)

Symmetry codes: (i) −*x*+2, −*y*+1, −*z*+1; (ii) −*x*+1, −*y*+1, −*z*+1; (iii) −*x*+1, −*y*+1, −*z*+2; (iv) −*x*+2, −*y*+1, −*z*+2; (v) *x*−1, *y*, *z*.

### Poly[isopropylammonium [dichloridozincate(II)]-µ-chlorido]] (IV) Crystal data

**Table:**

(C_3_H_10_N)[ZnCl_3_]	*F*(000) = 928
*M**_r_* = 231.84	*D*_x_ = 1.725 Mg m^−^^3^
Orthorhombic, *P**b**c**a*	Mo *K*α radiation, λ = 0.71073 Å
Hall symbol: -P 2ac 2ab	Cell parameters from 9690 reflections
*a* = 6.3131 (3) Å	θ = 5.8–59.0°
*b* = 12.9582 (6) Å	µ = 3.56 mm^−^^1^
*c* = 21.8277 (10) Å	*T* = 295 K
*V* = 1785.65 (14) Å^3^	Needle, colourless
*Z* = 8	0.47 × 0.22 × 0.19 mm

### Poly[isopropylammonium [dichloridozincate(II)]-µ-chlorido]] (IV) Data collection

**Table:**

Bruker D8 Quest Eco diffractometer	2642 reflections with *I* > 2σ(*I*)
φ and ω scans	*R*_int_ = 0.041
Absorption correction: multi-scan (SADABS; Krause *et al.*, 2015)	θ_max_ = 32.0°, θ_min_ = 3.7°
*T*_min_ = 0.444, *T*_max_ = 0.747	*h* = −9→9
137051 measured reflections	*k* = −19→19
3096 independent reflections	*l* = −32→32

### Poly[isopropylammonium [dichloridozincate(II)]-µ-chlorido]] (IV) Refinement

**Table:**

Refinement on *F*^2^	Hydrogen site location: mixed
Least-squares matrix: full	H atoms treated by a mixture of independent and constrained refinement
*R*[*F*^2^ > 2σ(*F*^2^)] = 0.036	*w* = 1/[σ^2^(*F*_o_^2^) + (0.0106*P*)^2^ + 2.9743*P*] where *P* = (*F*_o_^2^ + 2*F*_c_^2^)/3
*wR*(*F*^2^) = 0.076	(Δ/σ)_max_ < 0.001
*S* = 1.25	Δρ_max_ = 0.58 e Å^−^^3^
3096 reflections	Δρ_min_ = −0.74 e Å^−^^3^
88 parameters	Extinction correction: SHELXL-2018/3 (Sheldrick 2015b)
0 restraints	Extinction coefficient: 0.0049 (3)
Primary atom site location: dual	

### Poly[isopropylammonium [dichloridozincate(II)]-µ-chlorido]] (IV) Special details

**Table:**

Geometry. All esds (except the esd in the dihedral angle between two l.s. planes) are estimated using the full covariance matrix. The cell esds are taken into account individually in the estimation of esds in distances, angles and torsion angles; correlations between esds in cell parameters are only used when they are defined by crystal symmetry. An approximate (isotropic) treatment of cell esds is used for estimating esds involving l.s. planes.
Refinement. One low angle reflection with Fc>>Fo was presumed to have been blocked by the beam catcher and were omitted from the refinement. APEX3 control software suggested θmax = 37.20°, however data analysis showed that <I/σ(I)> is less than 1.64 for data beyond θ = 31.7°. Thus data with θ > 32.0° were omitted from the final refinement.

### Poly[isopropylammonium [dichloridozincate(II)]-µ-chlorido]] (IV) Fractional atomic coordinates and isotropic or equivalent isotropic displacement parameters (Å^2^)

**Table:**

	*x*	*y*	*z*	*U*_iso_*/*U*_eq_	
Zn1	0.71002 (4)	0.83231 (2)	0.19928 (2)	0.03189 (9)	
Cl1	0.69868 (10)	0.65958 (5)	0.19958 (3)	0.03814 (14)	
Cl2	0.58549 (13)	0.90289 (6)	0.11449 (3)	0.04834 (18)	
Cl3	0.56133 (10)	0.88709 (6)	0.29078 (3)	0.04376 (17)	
N1	0.6927 (5)	0.6086 (2)	0.34850 (11)	0.0427 (5)	
H1A	0.636 (7)	0.550 (4)	0.3524 (19)	0.076 (14)*	
H1B	0.622 (9)	0.655 (4)	0.327 (2)	0.105 (18)*	
H1C	0.803 (7)	0.601 (3)	0.326 (2)	0.077 (14)*	
C11	0.8781 (7)	0.5759 (3)	0.44385 (17)	0.0672 (10)	
H11A	1.004167	0.561033	0.420867	0.101*	
H11B	0.798477	0.51348	0.449539	0.101*	
H11C	0.916308	0.603761	0.483068	0.101*	
C12	0.7465 (6)	0.6526 (2)	0.40972 (14)	0.0516 (7)	
H12	0.832846	0.714465	0.403126	0.062*	
C13	0.5511 (8)	0.6841 (4)	0.44229 (17)	0.0870 (15)	
H13A	0.587641	0.712362	0.481553	0.13*	
H13B	0.461144	0.625124	0.447826	0.13*	
H13C	0.477813	0.735335	0.418599	0.13*	

### Poly[isopropylammonium [dichloridozincate(II)]-µ-chlorido]] (IV) Atomic displacement parameters (Å^2^)

**Table:**

	*U*^11^	*U*^22^	*U*^33^	*U*^12^	*U*^13^	*U*^23^
Zn1	0.02689 (14)	0.03437 (15)	0.03441 (15)	0.00108 (11)	−0.00115 (11)	0.00064 (11)
Cl1	0.0368 (3)	0.0321 (3)	0.0455 (3)	−0.0008 (2)	0.0005 (3)	−0.0010 (2)
Cl2	0.0528 (4)	0.0502 (4)	0.0420 (3)	0.0090 (3)	−0.0113 (3)	0.0061 (3)
Cl3	0.0264 (3)	0.0561 (4)	0.0488 (4)	−0.0090 (3)	0.0060 (2)	−0.0218 (3)
N1	0.0412 (13)	0.0533 (15)	0.0335 (11)	0.0061 (12)	0.0013 (10)	0.0031 (11)
C11	0.065 (2)	0.082 (3)	0.055 (2)	0.013 (2)	−0.0225 (18)	0.0027 (18)
C12	0.0607 (19)	0.0474 (16)	0.0465 (15)	0.0026 (15)	−0.0110 (15)	−0.0020 (13)
C13	0.099 (3)	0.113 (4)	0.049 (2)	0.052 (3)	−0.003 (2)	−0.020 (2)

### Poly[isopropylammonium [dichloridozincate(II)]-µ-chlorido]] (IV) Geometric parameters (Å, º)

**Table:**

Zn1—Cl1	2.2394 (7)	C11—H11B	0.96
Zn1—Cl2	2.2089 (7)	C11—H11C	0.96
Zn1—Cl3	2.3182 (7)	C11—C12	1.495 (5)
Zn1—Cl3^i^	2.3387 (7)	C12—H12	0.98
N1—H1A	0.84 (5)	C12—C13	1.481 (6)
N1—H1B	0.88 (6)	C13—H13A	0.96
N1—H1C	0.86 (5)	C13—H13B	0.96
N1—C12	1.492 (4)	C13—H13C	0.96
C11—H11A	0.96		
			
Cl1—Zn1—Cl2	113.89 (3)	H11A—C11—H11B	109.5
Cl1—Zn1—Cl3	106.89 (3)	H11A—C11—H11C	109.5
Cl1—Zn1—Cl3^i^	109.47 (3)	H11B—C11—H11C	109.5
Cl2—Zn1—Cl3	116.80 (3)	C12—C11—H11A	109.5
Cl2—Zn1—Cl3^i^	106.83 (3)	C12—C11—H11B	109.5
Cl3—Zn1—Cl3^i^	102.19 (2)	C12—C11—H11C	109.5
Zn1—Cl3—Zn1^ii^	111.78 (3)	C11—C12—C13	114.0 (3)
H1A—N1—H1B	116 (4)	C11—C12—H12	107.9
H1A—N1—H1C	108 (4)	C13—C12—H12	107.9
H1B—N1—H1C	100 (4)	C12—C13—H13A	109.5
C12—N1—H1A	111 (3)	C12—C13—H13B	109.5
C12—N1—H1B	110 (3)	C12—C13—H13C	109.5
C12—N1—H1C	112 (3)	H13A—C13—H13B	109.5
N1—C12—C11	108.6 (3)	H13A—C13—H13C	109.5
N1—C12—C13	110.2 (3)	H13B—C13—H13C	109.5
N1—C12—H12	107.9		

Symmetry codes: (i) *x*+1/2, *y*, −*z*+1/2; (ii) *x*−1/2, *y*, −*z*+1/2.

### Poly[isopropylammonium [dichloridozincate(II)]-µ-chlorido]] (IV) Hydrogen-bond geometry (Å, º)

**Table:**

*D*—H···*A*	*D*—H	H···*A*	*D*···*A*	*D*—H···*A*
N1—H1*A*···Cl2^iii^	0.84 (5)	2.47 (5)	3.292 (3)	165 (4)
N1—H1*B*···Cl1	0.88 (6)	2.81 (5)	3.317 (3)	118 (4)
N1—H1*B*···Cl1^ii^	0.88 (6)	2.74 (5)	3.356 (3)	128 (4)
N1—H1*C*···Cl1^i^	0.86 (5)	2.67 (5)	3.427 (3)	148 (4)
C12—H12···Cl2^i^	0.98	2.94	3.922 (4)	179

Symmetry codes: (i) *x*+1/2, *y*, −*z*+1/2; (ii) *x*−1/2, *y*, −*z*+1/2; (iii) −*x*+1, *y*−1/2, −*z*+1/2.
