# Supplementary material for: Iso­propyl­ammonium halidometallates. I. [CoX4]2−·X− (X = Cl, Br), ZnCl42−, and [ZnCl3−]n salts
Source: Acta Crystallogr E Crystallogr Commun. 2025 Jul 15;81(Pt 8):684–93. doi: 10.1107/S2056989025006103 (PMC12326503; doi:10.1107/S2056989025006103)

1. Histograms for C-C bond lengths (Å) in IPA^+^ for all structures (top) and only ordered structures (bottom).


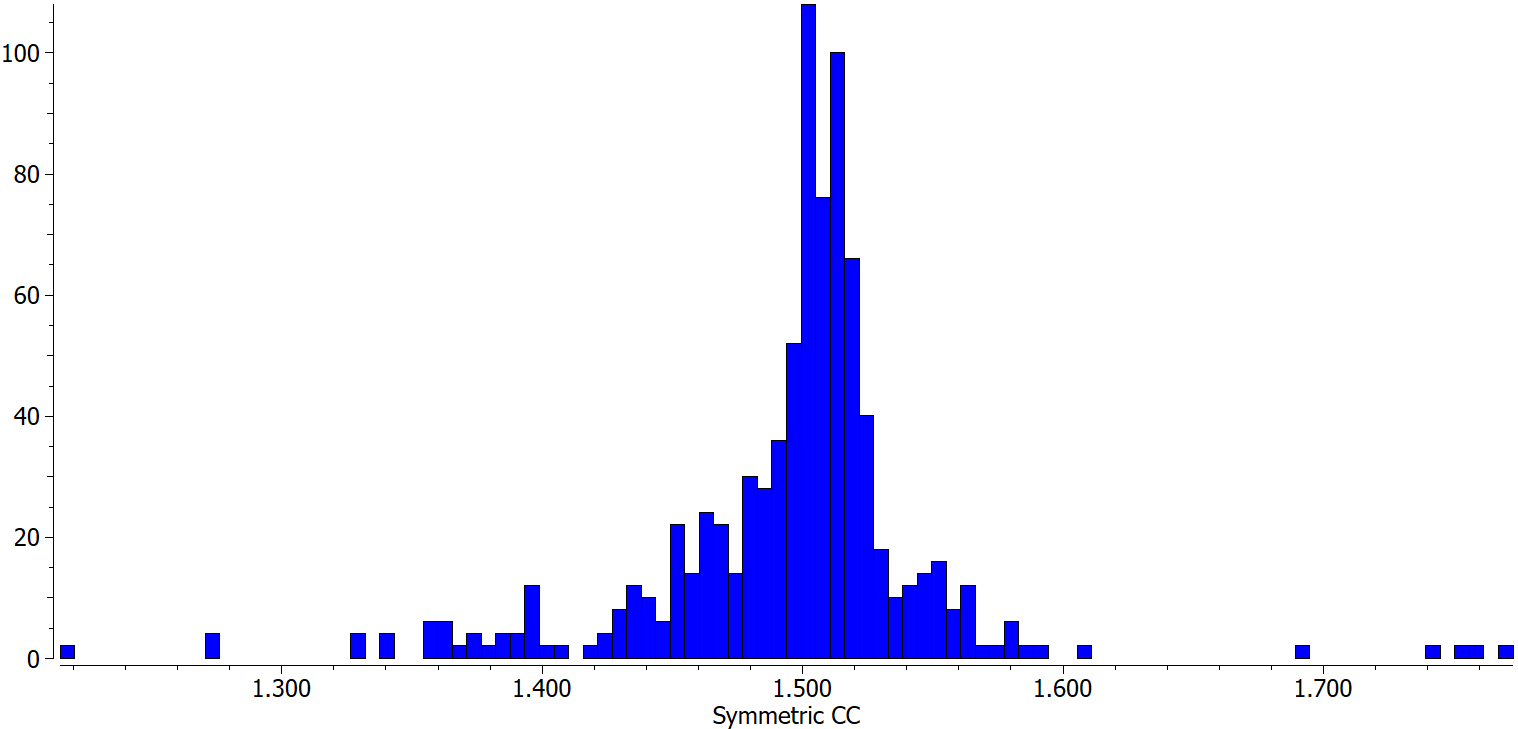


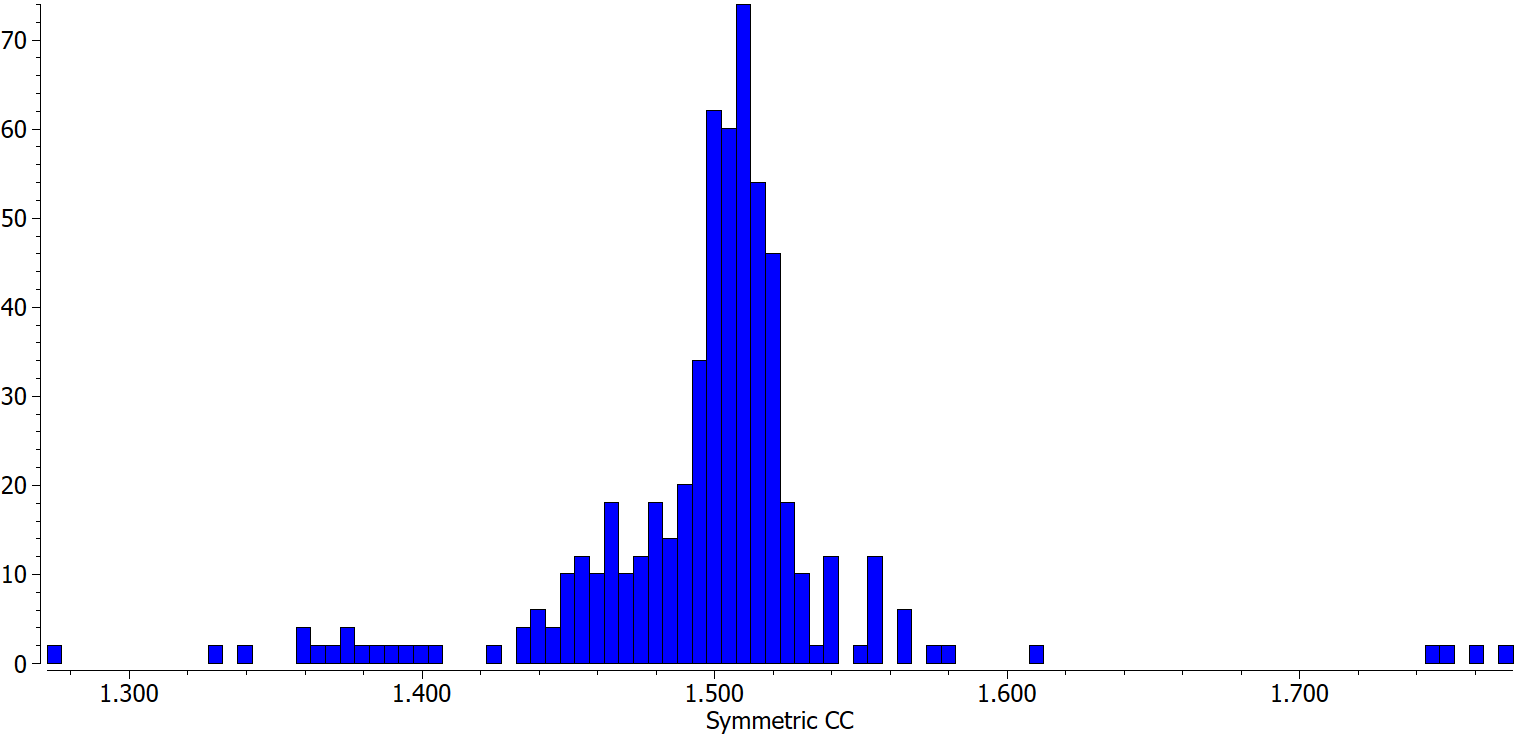


1. Histograms for C-N bond lengths (Å) in IPA^+^ for all structures (top) and only ordered structures (bottom).


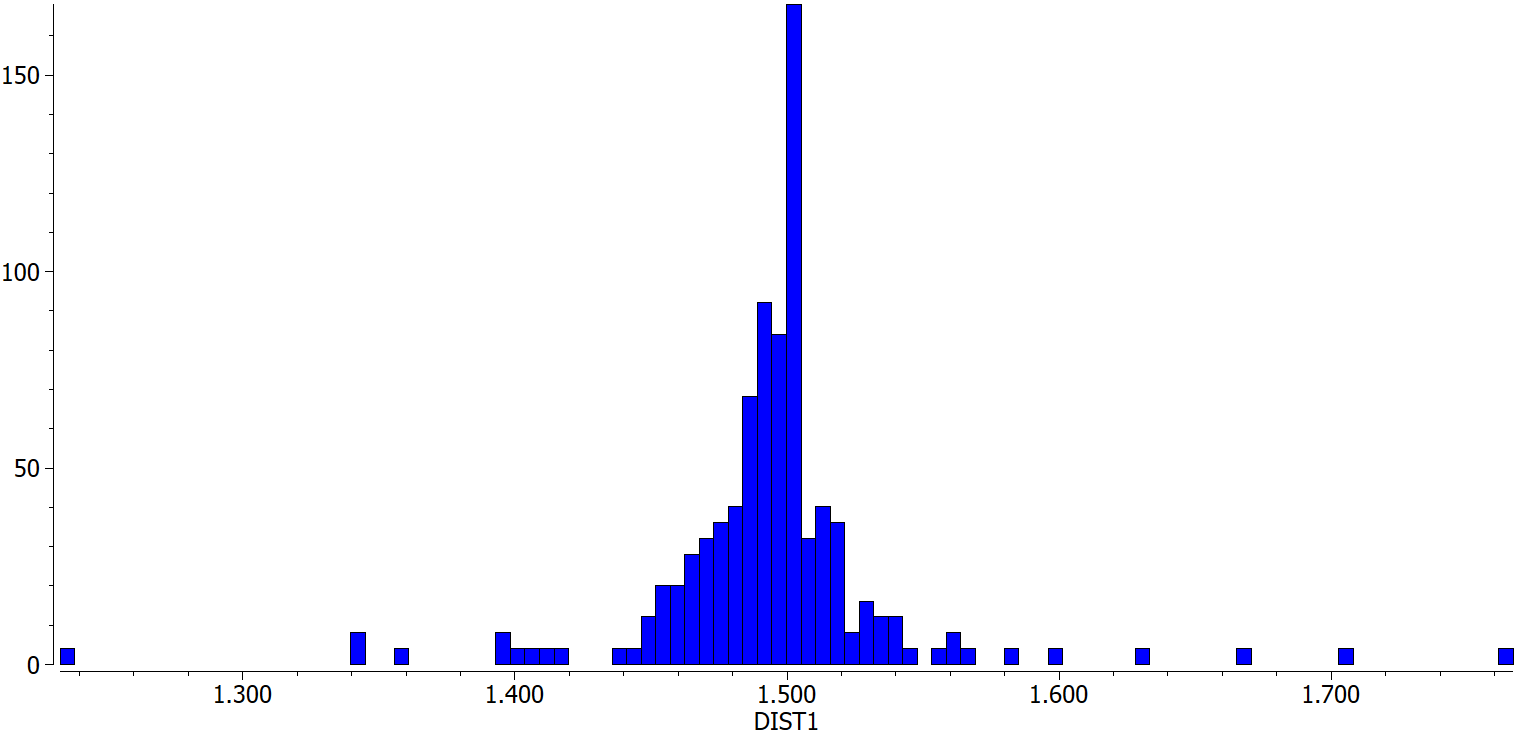


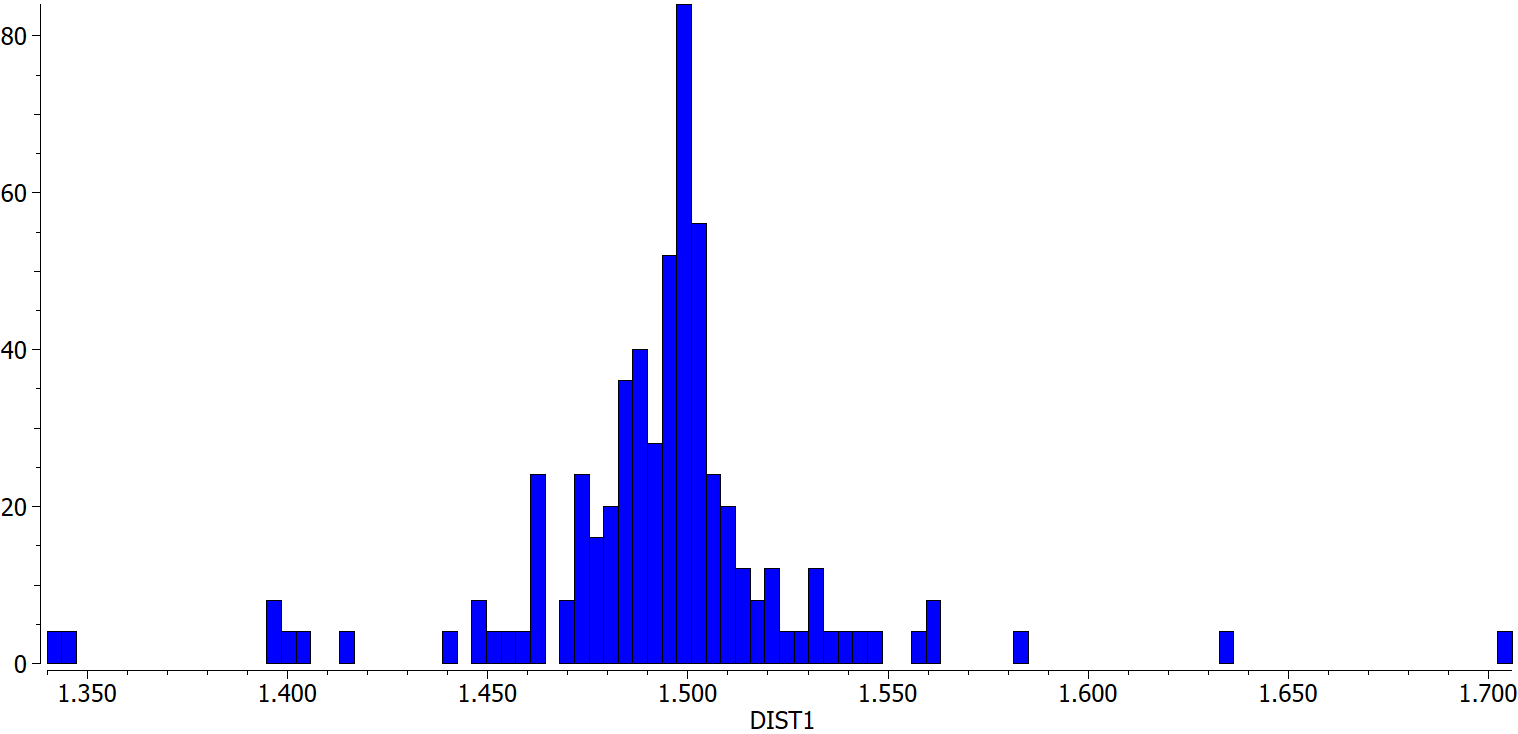


1. Histograms for C- C-N bond angles (°) in IPA^+^ for all structures (top) and only ordered structures (bottom).


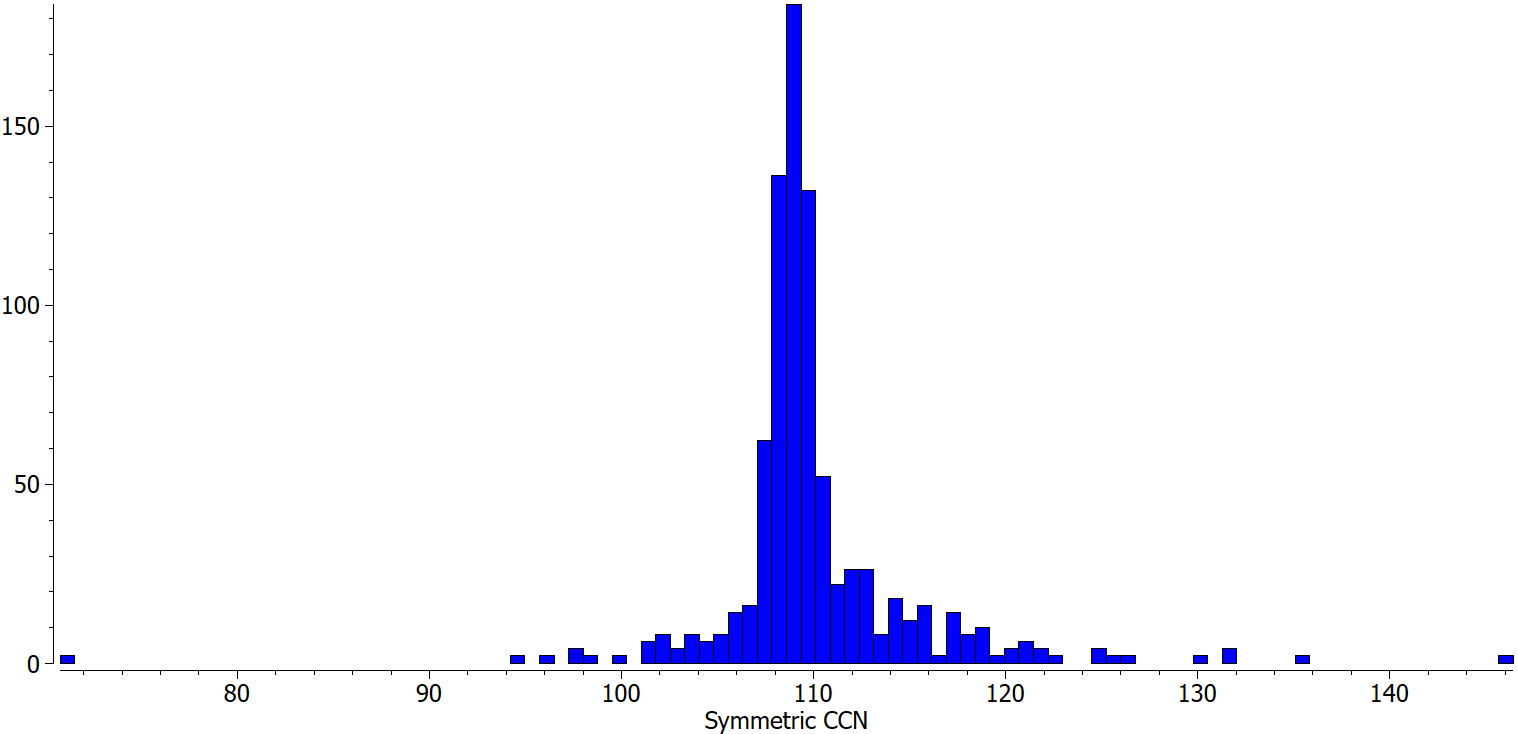


\


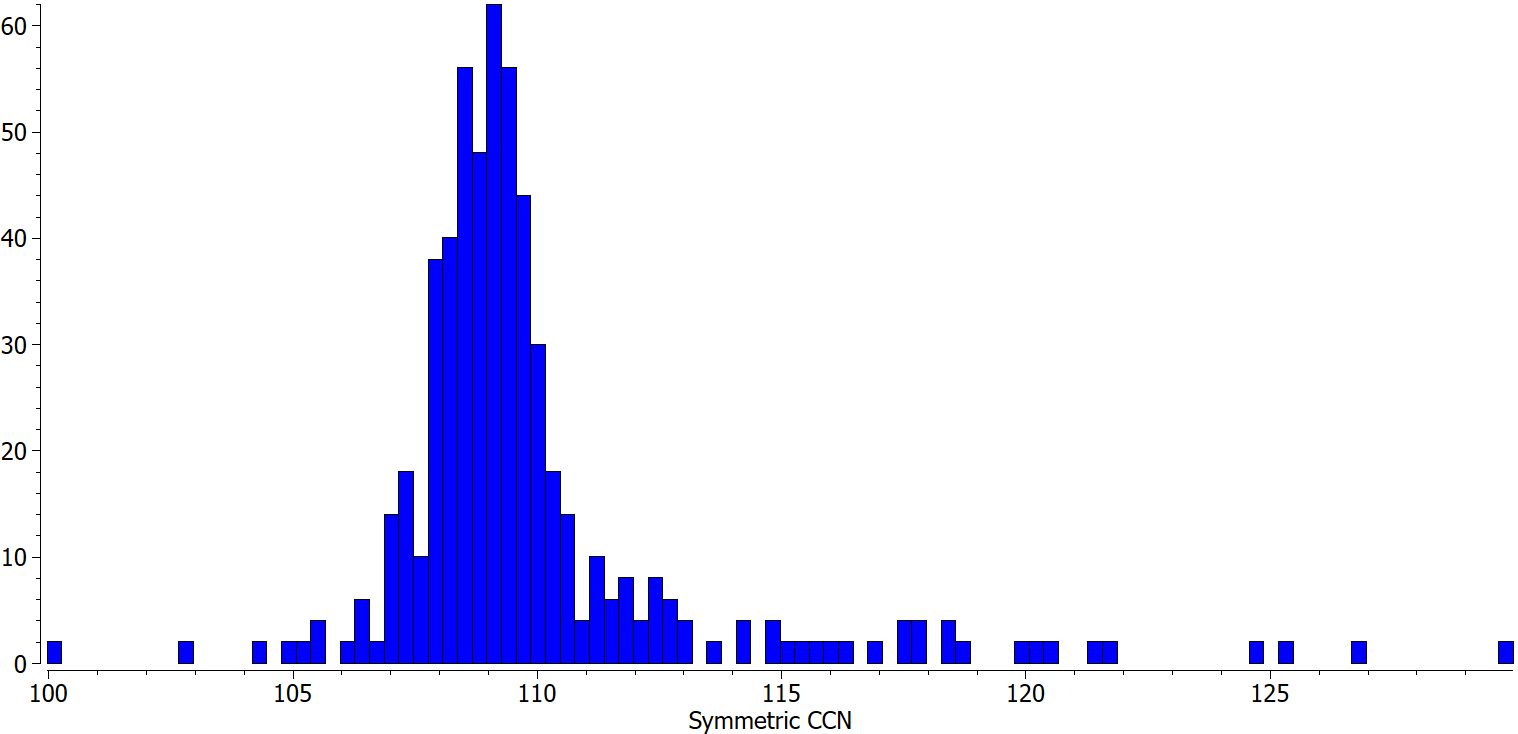


1. Histograms for C- C-C bond angles (°) in IPA^+^ for all structures (top) and only ordered structures (bottom).


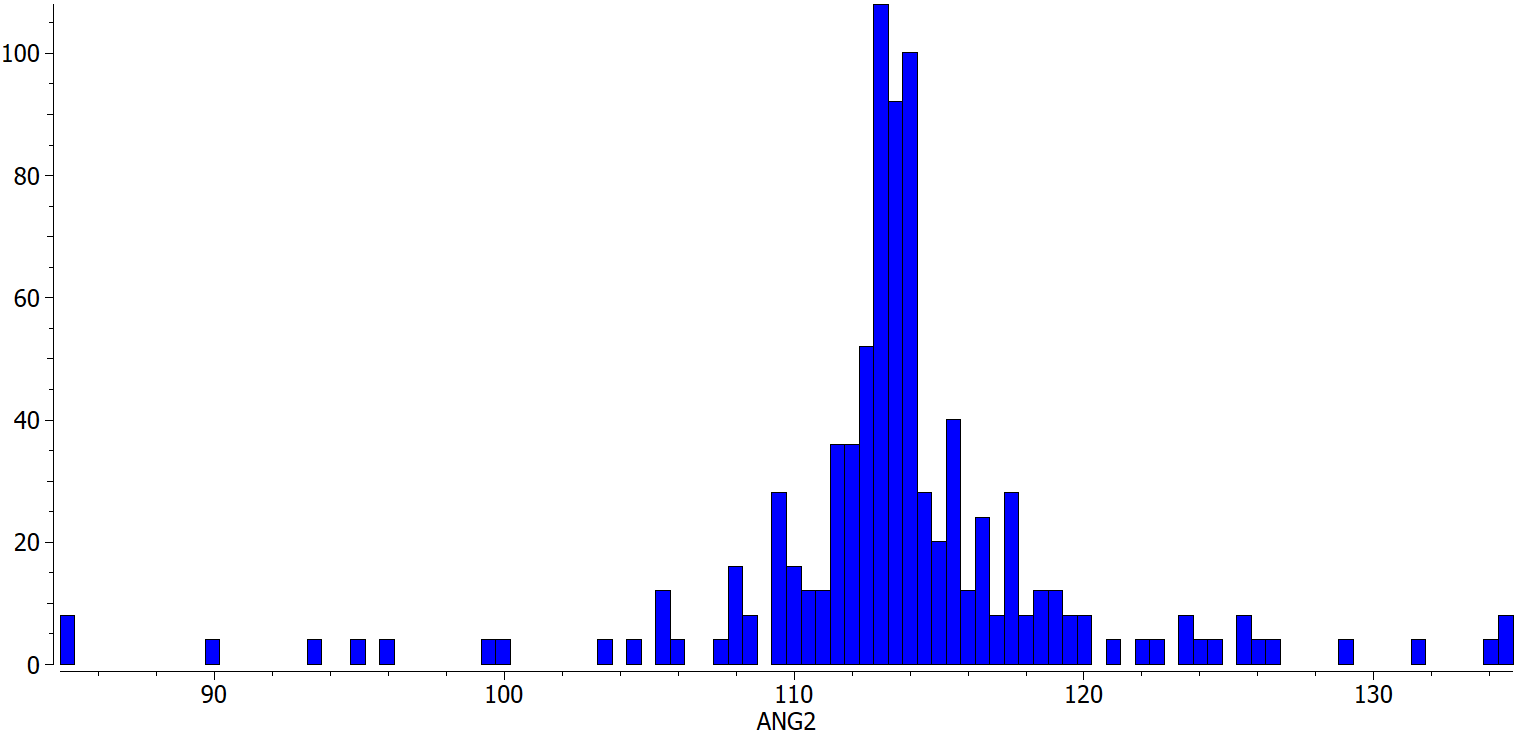


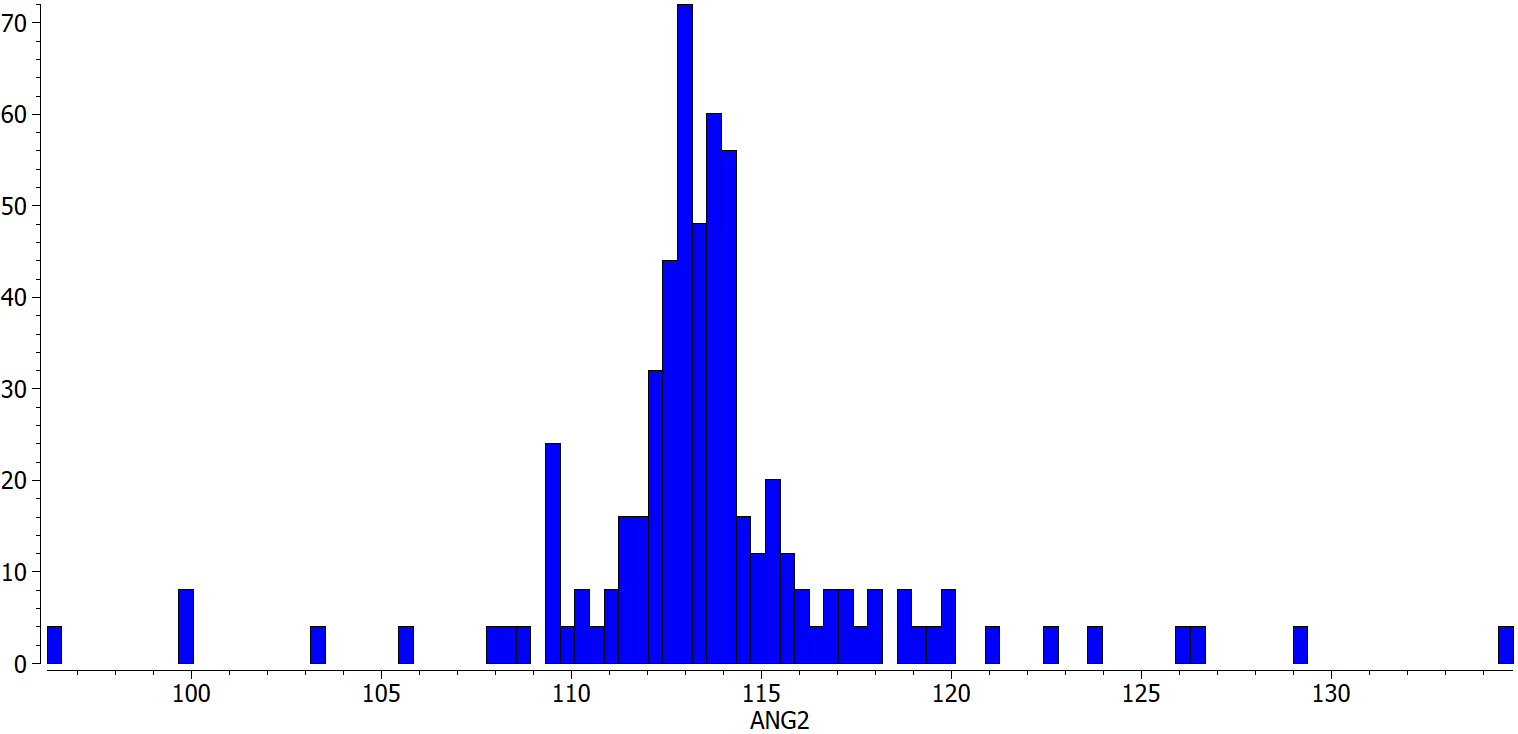


1. Histograms for C-N bond lengths (Å) in TMA^+^ for all structures (top) and only ordered structures (bottom).


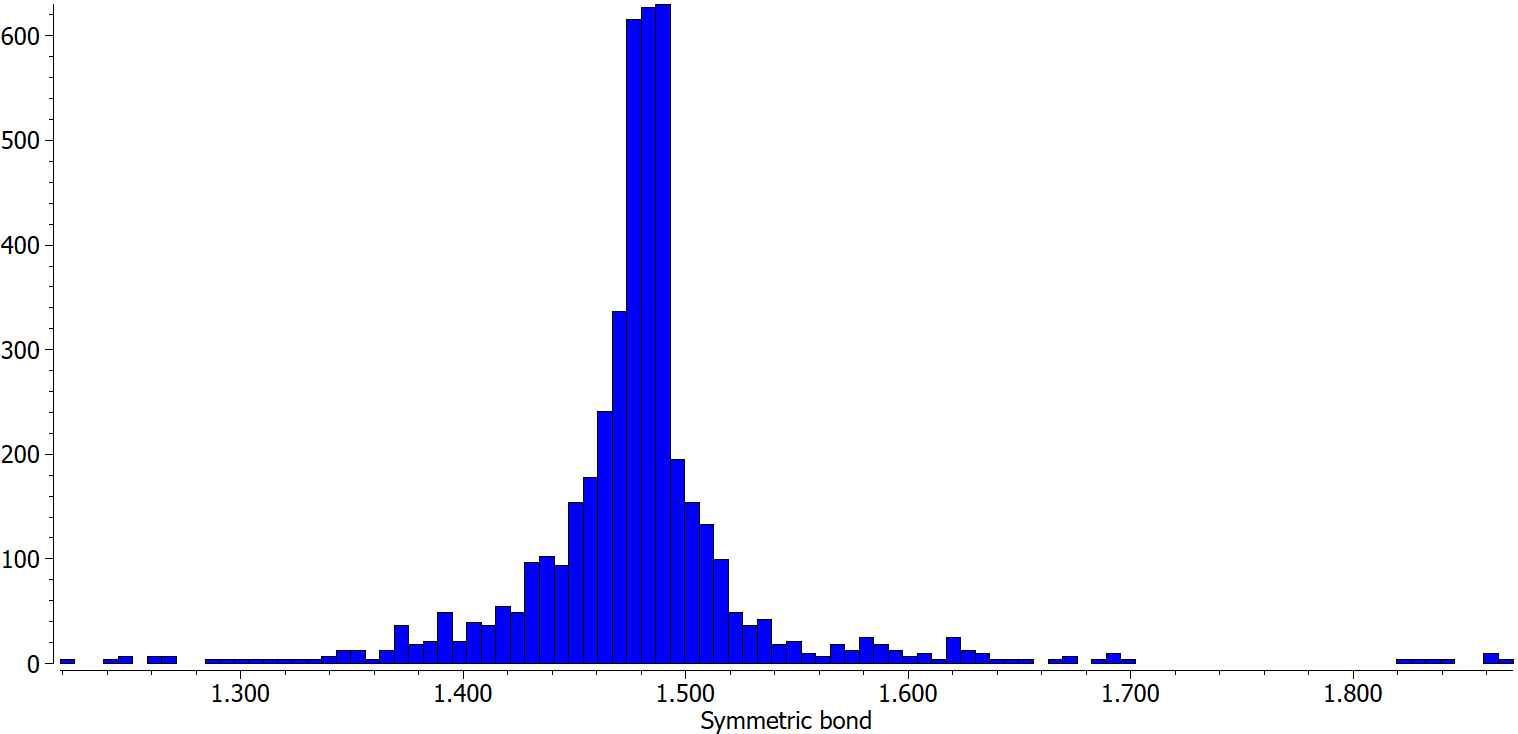


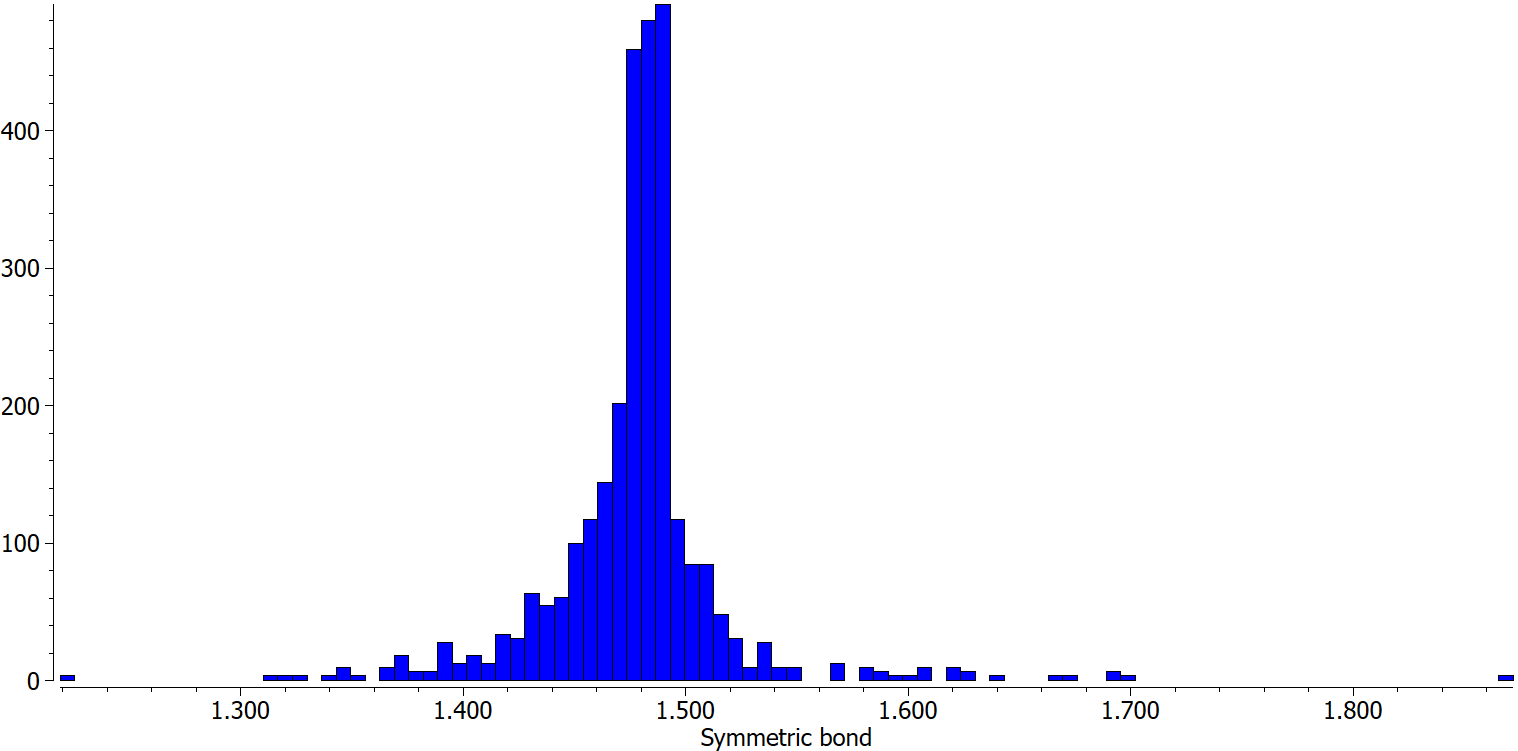


1. Histograms for C-N-C bond angles (°) in TMA^+^ for all structures (top) and only ordered structures (bottom).


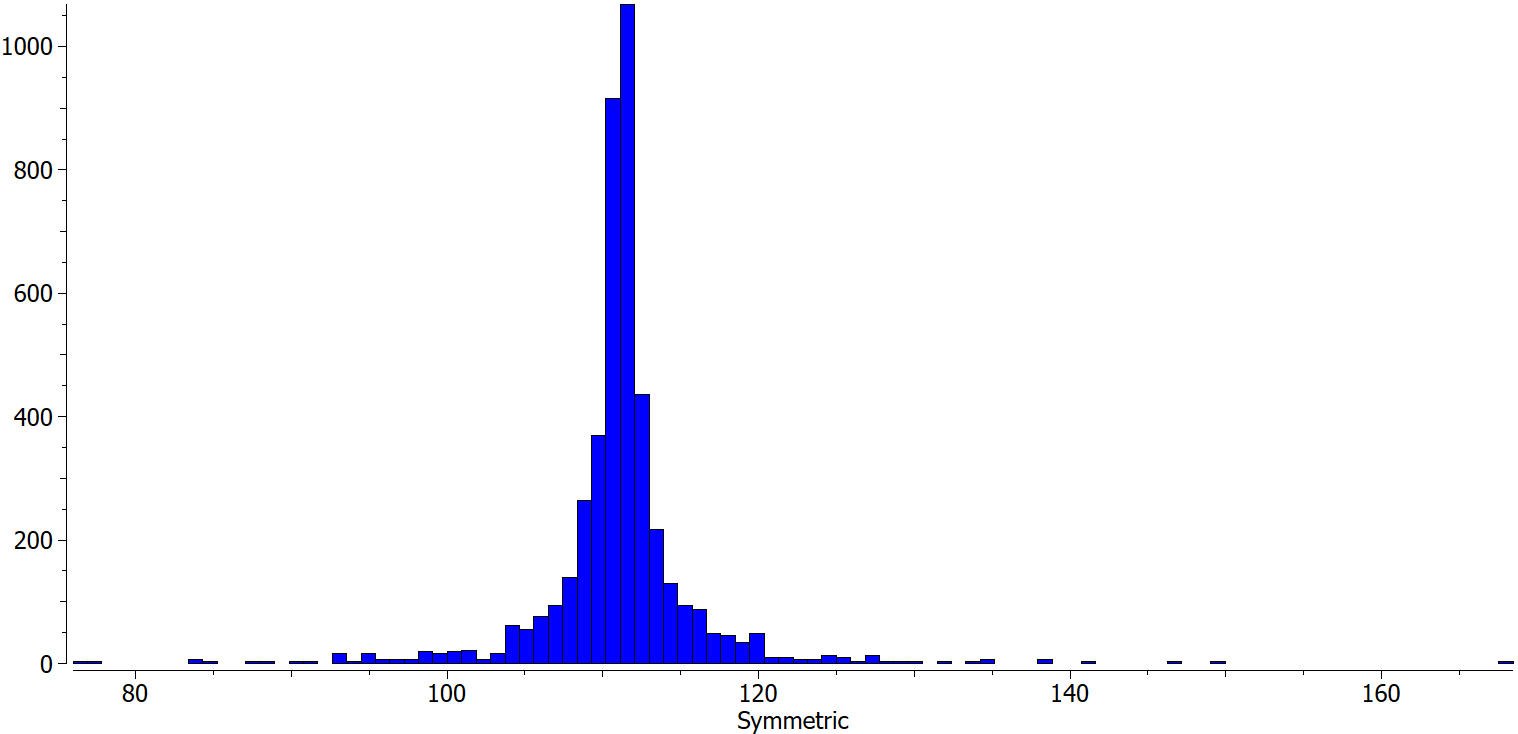


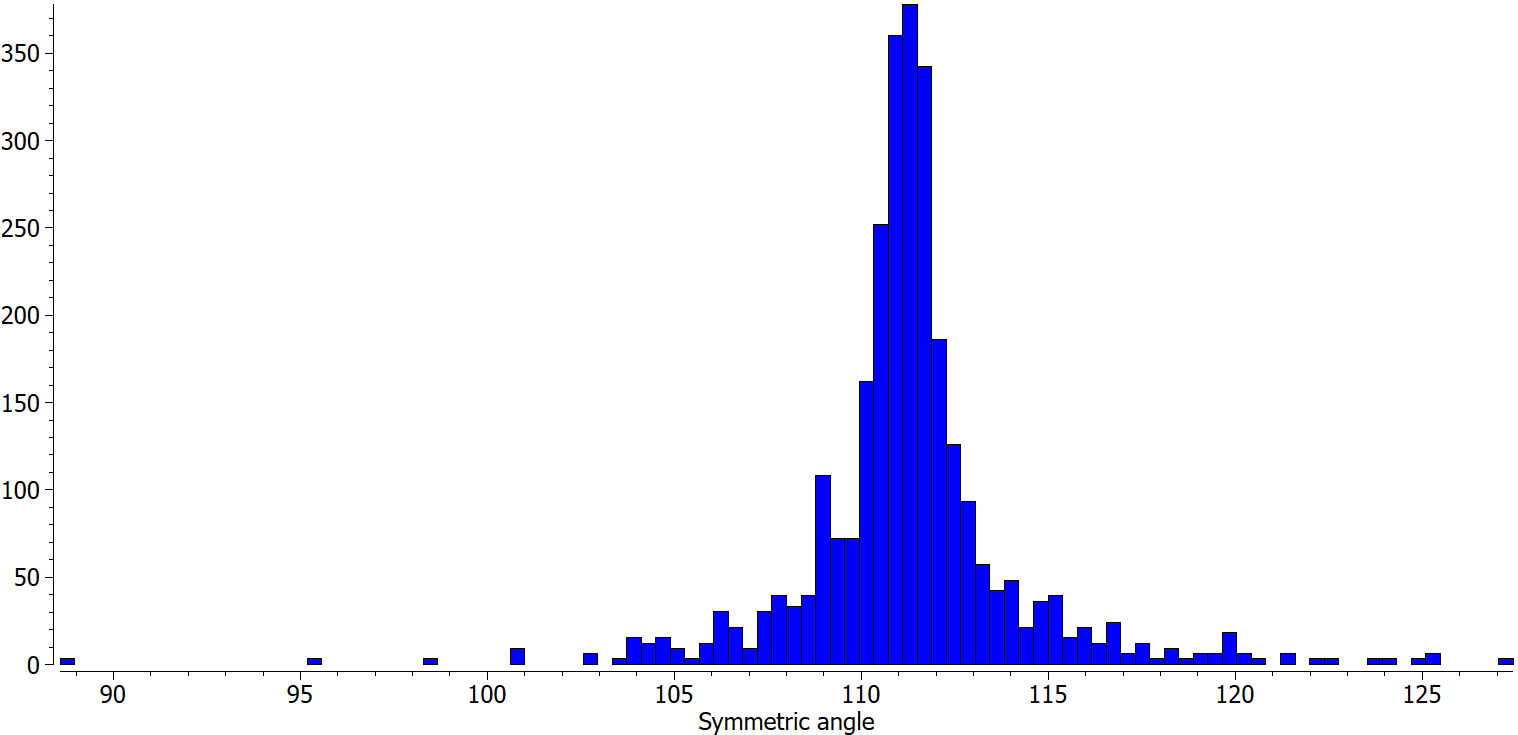

Supplement: Supplementary file 6 [file e-81-00684-sup6.docx]
