# Supplementary material for: Iso­propyl­ammonium halidometallates. I. [CoX4]2−·X− (X = Cl, Br), ZnCl42−, and [ZnCl3−]n salts
Source: Acta Crystallogr E Crystallogr Commun. 2025 Jul 15;81(Pt 8):684–93. doi: 10.1107/S2056989025006103 (PMC12326503; doi:10.1107/S2056989025006103)
Supplement: Supplementary file 7 [file e-81-00684-sup7.docx]

| Cation | A_2_CoCl_4_ Refcode | A_2_ZnCl_4_ Refcode | Crystal system and Spacegroup |
| --- | --- | --- | --- |
| triethylammonium | ACUGOW | QEZLEP | Orthorhombic Pbca |
| 3-chloropyridinium | ALAHUQ | NULRES | Triclinic P$\bar{1}$ |
| 3-bromopyridinium | ALAJAY | NULRIW | Triclinic P$\bar{1}$ |
| 4-(Me_2_N)pyridinium | APUQOR/BUZFIN | ULAQAA | Triclinic P$\bar{1}$ |
| 1-(n-Bu) pyridinium | BICDOK (Co/Zn) | WEQKAH | Monoclinic P2_1_/*n* |
| benzotriazolium | BISNOG | BISNIA | Monoclinic C2/*c* |
| 1,3,4-Me_3_pyridinium | COQZAM | COMGAP | Orthorhombic F*dd*2 |
| 2-aminopyridinium | DELPOA | WABHIS(01) | Monoclinic C2/*c* |
| 4-acetylaniliniuum | DUTMIQ | GOZVAU | Orthorhombic Cm*ca* |
| n-octylammonium | EMUMEH | QAWGAY | Monoclinic P2_1_/*n* |
| (3-Cl-2-OHpropyl)  trimethylammonium | ETONOT | UDAWEF | Monoclinic C2/*c* |
| pyridinium | EWAXIJ | WOHFII | Triclinic P$\bar{1}$ |
| 2-amino-4-methyl-  pyridinium | FAPBEH | VJURZAK (showing protonated amine) | Triclinic P$\bar{1}$ |
| *N*-Carbethoxymethyl-1,10-phenanthrolinium | FIKDUA | FIKFEM | Triclinic P$\bar{1}$ |
| chloromethyltrimethyl-  ammonium | GEYPAE  GEYPAE01 (P*n*m*a*)  GEYZAO (P2_1_2_1_2_1_) | FENCAF | Monoclinic P2_1_/*c* |
| 1-dodecyl-2,3-dimethyl- 3-imidazolium | GIWCAT | GIWCEX | Triclinic P$\bar{1}$ |
| N,N,N-trimethyl-  anilinium | GOLKUN | ZATNUD | Monoclinic C2/*c* |
| 2-amino-6-methyl- pyridinium | IXONER | GAKVIY | Triclinic P$\bar{1}$ |
| tetraphenylphosphonium | JOGXOU | OJUGOQ | Monoclinic C2/*c* |
| 1-(n-butyl)-3-methylimidazolium | KISMOP | KISMUV | Monoclinic C*c* |
| 2-methyl-3-(pyridin-2-yl)imidazo[1,5-a]pyridine-2-ium | LOJFUO (P2_1_/*n*)  V=2646 Å^3^  *Z*’=1 | HUMHII (P*bca*)  V=5240 Å^3^  *Z*’=1 | Dissimilar |
| Methadone | METHCO | METHZN | Triclinic |
| 2-chloropyridinium | MOGBEQ | LAVNUU(01) | Orthorhombic P*ccn* |
| 2,2'-Iminodipyridinium | NIPLAZ(01) | TOBHIC | Monoclinic C2/*c* |
| lignocaine | NIRTIR  *Z*’=2 | BEVMOE(01)  *Z*’=1 | Monoclinic P2_1_/*c*,  a axis doubled for Co |
| adamantylammonium | NIWMOW | EHIWEY | Monoclinic C2/m |
| Pyrazolium | NUZHUM | NUZJIC | Monoclinic P2_1_/*n* |
| Azetidinium | OFUFUU(01) (P*n*m*a*) OFUFUU02/03  (P2_1_/*n*) | QORXON (P2_1_/*n*)  QORXON01 (P*n*m*a*) | Monoclinic at ambient temperature, orthorhombic at high temperature |
| methylammonium | POKQUE | MAMZCL | P2_1_/*a* or P2_1_/*c* |
| 2-methylimidazolium | PUNDIN | VUSGIB | Monoclinic C2/*c* |
| 2-(4,6-di-t-butylpyridin-1-ium-2-yl)-N,N'-dihexylmalonamide | QORNOB | QORNUH(01) | Tetragonal I4_1_/*a* |
| tetraethylammonium | QQQALM | TEKYAJ  TEKYAJ01  (C2*ca*, 190 K) | Tetragonal P4_2_/*n*m*c* |
| 2-amino-5-bromo-pyrimidinium | RENNAA | RENNEE | Triclinic P$\bar{1}$ |
| guanadinium | RUMRUM | HORGOK(01,02) | Triclinic P$\bar{1}$ |
| 2-amino-5-methyl-pyridinium | SUTSIK | TAHMOF | Monoclinic P2_1_/c |
| dimethylammonium | SINSUD(01-08)  SINSUD04-phase transition (235 K) w/retention of space grouo | SINSOX(01-04) | Monoclinic P2_1_/*n* |
| tetramethylammonium | TMACCO(01) | TMACZN(01-09)  Lower symmetry phases at low T | Orthorhombic P*n*m*a* |
| 1-(2,6-di-isopropylphenyl)-2,2,4,4-tetramethyl-3,4-dihydro-2H-pyrrolium | TOCFAU  Orthorhombic P2_1_2_1_2_1_ | WEFOG  Triclinic P$\bar{1}$ | Dissimilar |
| 8-methylquinolinium | UFAPUO | ILEFEL | Triclinic P$\bar{1}$ |
| *N*-(4-picolinium)-1,8-naphthalimide | UFOXEV | UFOXOF | Triclinic P$\bar{1}$ |
| 2-amino-5-chloro-pyridinium | UHUHEM | ULASAC | Monoclinic P2_1_/c |
| 8-hydroxyquinolinium | UQIHIN(01,02) | FARFIP(01,02) | Monoclinic C2/c |
| 2-phenylethylammonium | UQUYIP | EYUWAX | Monoclinic P2_1_/c |
| novocaine | VILDEZ | VOHKAE | Monoclinic B2/*b* |
| tetramethylarsonium | VISZAY(01)  (I4_1_/a at high T) | VISYUR | Tetragonal P4_2_/m*bc* |
| 2-chloro-N,N-dim ethylethan-1-aminium | VOTDUH | VOTFAP | Monoclinic C2/c |
| lidocaine | WACZUV | BEVMOE | Monoclinic P2_1_/c |
| 1-dodecylpyridinium | WEQKEL | WEQKIP | Triclinic P$\bar{1}$ |
| 1-butylpyridinium | WEQKUB | WEQKAH | Monoclinic P2_1_/*n* |
| 4-[bis(2-cyanoethyl)-amino]pyridinium | XINMAK | XINVEX | Orthorhombic P*na*2_1_ |
| 2,6-dimethylpyridinum | YIZSEF(01,02) | XUCNUG | Orthorhombic P*bcn* |
| 3-(butylamino)-N,N-diisobutyl-3-oxopropan-1-aminium | YUPJAW | YUPJEA | Monoclinic P2_1_/*c* |
| triphenylmethylarsonium | ZZZWOW | ZZZWOY | Cubic P2_1_3 |
